# Simultaneous fMRI-EEG-DTI recording of MMN in patients with schizophrenia

**DOI:** 10.1371/journal.pone.0215023

**Published:** 2019-05-09

**Authors:** Eiji Kirino, Yayoi Hayakawa, Rie Inami, Reiichi Inoue, Shigeki Aoki

**Affiliations:** 1 Department of Psychiatry, Juntendo University Shizuoka Hospital, Izunokuni City, Shizuoka, Japan; 2 Department of Psychiatry, Juntendo University School of Medicine, Hongo, Bunkyo-ku, Tokyo, Japan; 3 Juntendo Institute of Mental Health, Fukuroyama, Koshigaya City, Saitama, Japan; 4 Department of Radiology, Graduate School of Medicine, University of Tokyo, Bunkyo-ku, Tokyo, Japan; 5 Department of Radiology, Juntendo University School of Medicine, Hongo, Bunkyo-ku, Tokyo, Japan; Universidad de Salamanca, SPAIN

## Abstract

Functional magnetic resonance imaging (fMRI), electroencephalogram (EEG), and diffusion tensor imaging (DTI) recording have complementary spatiotemporal resolution limitations but can be powerful methods when used together to enable both functional and anatomical modeling, with each neuroimaging procedure used to maximum advantage. We recorded EEGs during event-related fMRI followed by DTI in 15 healthy volunteers and 12 patients with schizophrenia using an omission mismatch negativity (MMN) paradigm. Blood oxygenation level-dependent (BOLD) signal changes were calculated in a region of interest (ROI) analysis, and fractional anisotropy (FA) in the white matter fibers related to each area was compared between groups using tract-specific analysis. Patients with schizophrenia had reduced BOLD activity in the left middle temporal gyrus, and BOLD activity in the right insula and right parahippocampal gyrus significantly correlated with positive symptoms on the Positive and Negative Syndrome Scale (PANSS) and hostility subscores. BOLD activation of Heschl’s gyri also correlated with the limbic system, including the insula. FA values in the left anterior cingulate cortex (ACC) significantly correlated with changes in the BOLD signal in the right superior temporal gyrus (STG), and FA values in the right ACC significantly correlated with PANSS scores. This is the first study to examine MMN using simultaneous fMRI, EEG, and DTI recording in patients with schizophrenia to investigate the potential implications of abnormalities in the ACC and limbic system, including the insula and parahippocampal gyrus, as well as the STG. Structural changes in the ACC during schizophrenia may represent part of the neural basis for the observed MMN deficits. The deficits seen in the feedback/feedforward connections between the prefrontal cortex and STG modulated by the ACC and insula may specifically contribute to impaired MMN generation and clinical manifestations.

## Introduction

Functional magnetic resonance imaging (fMRI) and electroencephalogram (EEG) imaging have complementary spatiotemporal resolution limitations and can be powerful methods when used together to understand brain function. However, functional connectivity analyses using fMRI and EEG require anatomical model-based hypotheses. Diffusion tensor imaging (DTI) measures the diffusion direction of water molecules, allowing the visualization of axonal projections in the white matter and fiber connectivity. Adding DTI to simultaneous magnetic resonance imaging (MRI) and EEG recordings enables both functional and anatomical modeling, with each neuroimaging procedure used to maximum advantage.

Mismatch negativity (MMN) is an event-related potential (ERP) component generated by neuronal mismatch between the sensory memory input from a deviant auditory stimulus and a standard, a memory trace of frequent auditory stimuli [1]. Reduction in MMN in schizophrenia is a candidate biological marker [2]. MMN is elicited even when subjects are instructed to ignore the auditory stimulation, suggesting that it may be an automatic process [1, 3–7]. MMN has been hypothesized to be an automatic alerting mechanism that stimulates individuals to explore unexpected environmental events [8], with a fronto-central scalp distribution pattern that can be modeled by generator sources in anterior regions of the superior temporal auditory cortex [1]. An additional MMN generator has been identified in the frontal cortex, indicating that fronto-temporal projections or fronto-temporal feedback processes may be involved in efficient MMN generation [9].

In source localization studies, a four-source model fitted to the MMN waveform was found in the left superior temporal gyrus (STG), anterior cingulate cortex (ACC), right STG, and inferior/mid frontal cortices [10]. Two dipoles in the auditory cortex and two in the frontal lobe (left cingulate and right inferior frontal cortex) were also found for pitch and duration MMN [11]. Spatiotemporal source imaging indicated additional generators within the ACC and the right inferior temporal gyrus that were clearly separated from the STG generators [12].

Mismatch responses for deviant stimuli in the primary and secondary auditory areas [13], superior temporal [14, 15] and inferior frontal cortices [16], or neostriatum [14] have been reported in fMRI studies in healthy subjects. The prefrontal cortex, particularly the mid-ventrolateral prefrontal cortex partially located in the inferior frontal gyrus (IFG), has been reported to contain the involuntary attention switch for auditory change, allocation of attentional resources, mediation of response inhibition, and updating the prediction model [9]. Forward and backward STG connections in the right IFG are thought to be involved in establishing and modulating the prediction model; a pre-attentive reorienting response in the right frontal lobe is triggered by the detection of bilateral auditory changes in the primary auditory cortex, which then feeds back to the auditory sensory regions [17].

The deviant stimulus is compared to representations of invariance across tones rather than representations of the individual tones. Invariance is defined as regular features or relationships between tones [18]. MMN can be elicited by tone pips in the shorter silent intervals, and MMN is clearly elicited by stimulus omission in a sequence of regularly spaced tones [19]. Therefore, MMN also seems to be generated due to something other than new afferent elements activated by deviant, non-standard stimuli [5].

Recent DTI studies have reported structural abnormalities in several fiber tracts in schizophrenia, confirming a disruption in the structure of the white matter connecting gray matter regions [20–24]. Disruptions between the cingulate cortex and other regions were demonstrated previously [25–28] and may be associated with the symptoms of schizophrenia [29–31]. Neuroimaging studies have also implicated the cingulate cortex, particularly the ACC, as one of the sources of MMN [10–12].

In the present study, we recorded EEGs during event-related fMRI followed by DTI to investigate the differences of MMN responses elicited by auditory stimulus omission between patients with schizophrenia and healthy controls. We examined the correlation between DTI, MMN, and fMRI to clarify the association between the phenotypes reflected by fMRI and EEG and the structural abnormalities revealed by DTI in patients with schizophrenia, focusing on the ACC in the DTI analysis. The connectivity of the ACC is thought to be disrupted in schizophrenia, and it is expected to contribute to MMN regulation by mediating the interaction between the temporal and frontal cortices.

## Methods

### Ethical statement

This study was approved by the Juntendo University Koshigaya Hospital Ethics Committee and Juntendo University Shizuoka Hospital Ethics Committee.

### Subjects

Twelve patients with schizophrenia (10 men and 2 women, mean age 36.8 ± 6.0 years, all right-handed except for one left-handed patient) and 15 healthy controls (13 men and 2 women, mean age 28.4 ± 9.6 years, all right-handed except for one left-handed subject) were included in the study. The groups were not age-matched (F[1,25] = 5.3, *p* = 0.11). Attempts were made to match the groups for years of education, but this proved to be difficult because patients with schizophrenia are more likely to require hospitalization and the early age of onset [32], which can interfere with schooling. Therefore, controls had significantly more (F[1,25] = 0.130, *p* < 0.001) years of education (17.3 ± 1.9) than patients (13.3 ± 2.0). The statistical procedures were adjusted for age and education. Patients were recruited from among the outpatients at Juntendo University Koshigaya Hospital in Saitama and Juntendo University Shizuoka Hospital in Shizuoka, Japan, and healthy controls were recruited from the staff of the same hospitals. All controls were healthy and native to Japan. The mean disease duration in the patient group was 14.3 ± 5.7 years, and the mean drug dose used for treatment (risperidone equivalent) was 10.5 ± 6.2 mg (Table 1). All patients were determined to be in good physical health based on medical history, physical examination, and laboratory measures. None of the subjects had a history of electroconvulsive shock treatment, alcohol or other drug abuse (DSM-5 criteria) [33], addiction, or a neurological illness affecting the central nervous system, and all subjects reported having normal visual acuity. The DSM-5 [33] diagnosis and Positive and Negative Syndrome Scale (PANSS) [34] score were determined for each patient using a structured psychiatric interview and by reviewing the patients’ medical charts. The PANSS is a semi-structured interview with a 7-point scale to rate 30 symptoms common in schizophrenia and other mental disorders. The mean PANSS scores were 15.4±6.9 (positive scales), 22.8±9.2 (negative scales), 36.8±10.3 (general psychopathology scales), and 74.9±22.9 (total score). All participants were informed of the objectives and methods of the study and given written informed consent before the study began. The treating psychiatrists evaluated the capability of the patients to give informed consent and confirmed that they understood the voluntariness of their participation. Any participants who lacked adequate understanding of the study were excluded.

**Table 1:** Profile of sample groups.

	Controls	Patients	Statistics
N	15	12	
Age	28.4±9.6	36.8±6.0	F(1,25) = 5.3, p = 0.11
Gender (male/female)	13/2	10/2	χ^2^ (1) = 1.707, p = 0.809
Handedness (left/right)	1/14	1/11	χ^2^ (1) = 2.286, p = 0.869
Duration of education (years)	17.3±1.9	13.3±2.0	F(1,25) = 0.130, p < 0.001
Duration of illness (years)	-	14.3±5.7	-
Dose of antipsychotic medication/chlorpromazine equivalent(mg)	-	1051.5±619.7	-
PANSS positive symptom subscale	-	15.4±6.9	-
PANSS negative symptom subscale	-	22.8±9.2	-
PANSS general psychopathology subscale	-	36.8±10.3	-
PANSS total score	-	74.9±22.9	-

### Five-component model of the PANSS

As described previously [35], clinical manifestations were evaluated by the PANSS [34], which is classified into five components according to the five-component model [36, 37]: negative (passive withdrawal, emotional withdrawal, blunted affects, lack of spontaneity, poor rapport, disturbance of volition, preoccupation, motor retardation), positive (delusions, unusual thoughts, somatic concern, grandiosity, suspiciousness, hallucinations), cognitive (difficulty in abstract thinking, stereotyped thinking, cognitive disorganization, lack of judgment and insight, poor attention, tension, mannerisms and posturing), emotional discomfort (depression, anxiety, guilt, active social avoidance), and hostility (excitement, hostility, impulse control, uncooperativeness).

### Auditory stimuli: Passive oddball-omission paradigm

ERPs and fMRI were recorded during a sound omission MMN paradigm in which repetitive sequences of a frequent auditory stimulus were omitted occasionally. A computer with custom-designed software generated the acoustic stimuli and controlled stimulus timing and presentation. Tones were presented binaurally at a constant listening level (92–95 dB sound pressure level) through tube-connected headphones held in place by a headset.

The acoustic stimuli consisted of sine wave tones with a duration of 35 ms, including 0 ms rise and 5 ms fall times. The tone frequency was 3000 Hz, and the onset-to-onset interval was 136 ms. The experimental task consisted of a single block including 6547 tones and 53 omission events. The probability of omission was 0.00803 (53/6600), and omission occurred in the 19^th^ location in the sound trains during the “event” scans.

ERPs were recorded in a ‘passive’ condition in which the subjects were asked to ignore the stimuli and watch a silent movie projected on a video monitor. All subjects were told that they would have to give specific feedback about the movie at the end of the ERP session to standardize their level of attention. Subjects were also instructed to avoid unnecessary eye movement and blinking during the session.

The 136-ms stimulus onset asynchrony (SOA) of the acoustic stimuli used in the present study was selected to be within the temporal window of integration (TWI) proposed by Yabe et al. [19, 38]. Acoustic signals presented within an approximate 200 ms temporal window are integrated into a single perceptual unit [39]. In addition, Yabe et al. [19, 40] showed that acoustic information is integrated into a single unit within 160–170 ms to form a sound trace in sensory memory via a stimulus-omission MMN paradigm. TWI is an aspect of temporal integration that determines the initial sound energy unit in the early, pre-attentive stages of auditory information processing [19, 40].

### fMRI design and synchronization between scan noise and auditory stimuli

**Fig 1:**
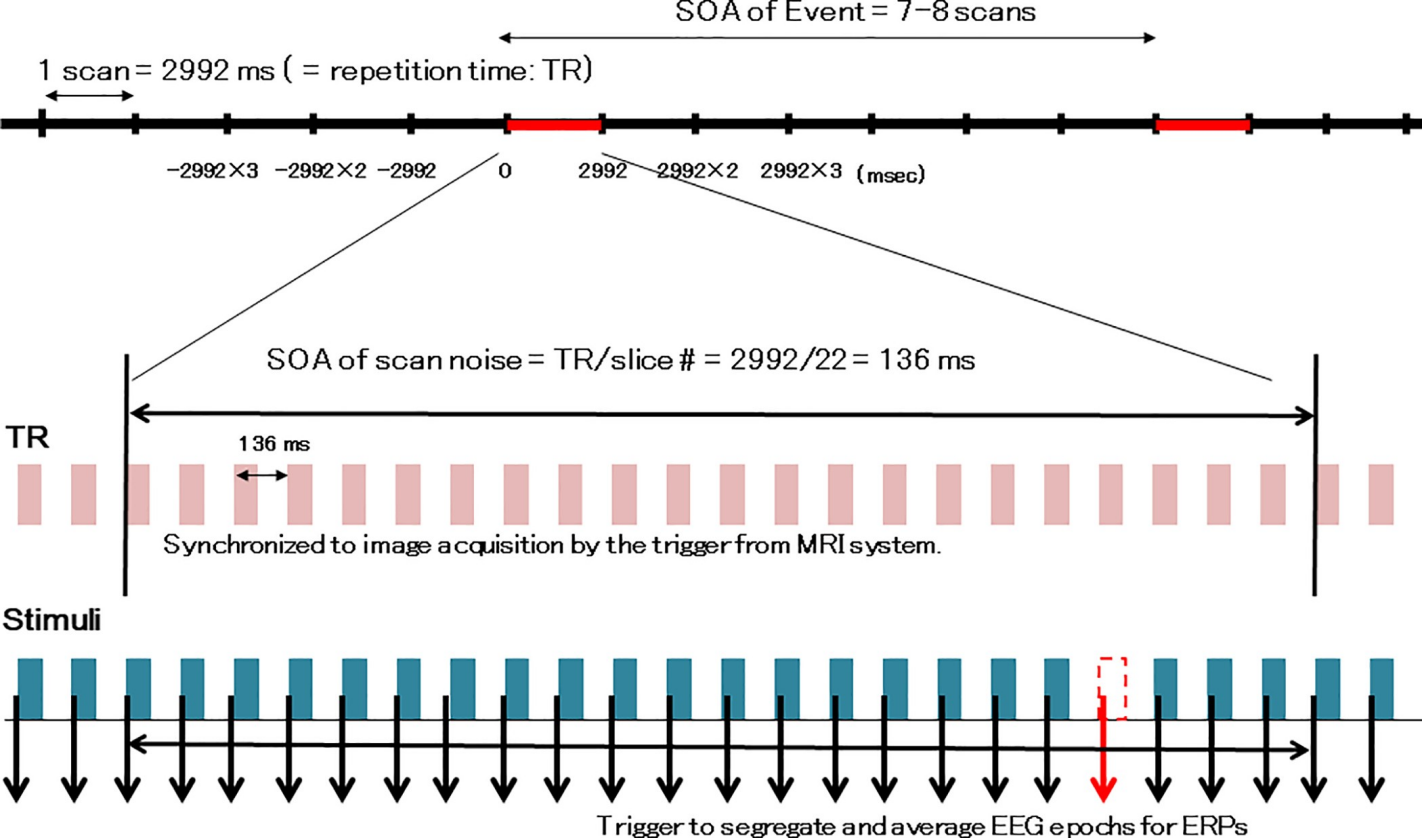
Schematic of functional magnetic resonance imaging (fMRI) data acquisition using an event-related design. We used an event-related design for the fMRI. Scans of 22 slices generated equal numbers of scan noises during a single repetition time (TR) of 2992 ms, and the same number of tones with a stimulus onset asynchrony (SOA) of 136 ms (TR/slice number = 2992/22) were presented. The presentation of tones was completely synchronized to scans by triggers generated by the MRI system in order to minimize the effect of scan noise on fMRI and electroencephalogram (EEG) raw data, which were also used to segregate and average EEG epochs in the ERP analysis. The SOA for event scans including the omission events and tones in the regular positions represent 7 or 8 scans (20,944 or 23,936 ms). Fifty-three event scans were assorted at random among the 247 baseline scans including baseline tones only, with no omission. Total scan time = 2992 ms x 300 scans = 897,600 ms. Event: scan including omission 53 times, indicated by red crossbars. Baseline: scan including only frequent stimuli 247 times, indicated by black crossbars. Frequent stimuli: 6547 trials, 3000 Hz, duration 35 ms, rise/fall 0/5 ms. Rare (omission) events: 53 trials (probability: 53/6600 = 0.00803).

We used an event-related design for the fMRI. Scans of 22 slices generated equal numbers of scan noises during a single repetition time (TR) of 2992 ms, and the same number of tones with an SOA of 136 ms (TR/slice number = 2992/22) were presented. The presentation of tones was completely synchronized to scans by triggers generated by the MRI system in order to minimize the effect of scan noise on fMRI and EEG raw data, which were also used to segregate and average EEG epochs in the ERP analysis. We averaged EEG epochs and subtracted the waveforms of the baseline tones from the omission events during ERP processing to eliminate fluctuations in the EEG signals due to scan noise. A similar concept was applied for blood oxygen level-dependent (BOLD) signal processing using an event-related fMRI design. The SOA for event scans including the omission events and tones in the regular positions represent 7 or 8 scans (20,944 or 23,936 ms). Fifty-three event scans were assorted at random among the 247 baseline scans including baseline tones only, with no omission (Fig 1). BOLD hemodynamic responses for event scans were then compared to those for baseline scans.

### Image acquisition for fMRI

BOLD fMRI data acquisition was performed at Juntendo University Hospital using a Philips Achieva 3.0 Tesla MRI scanner with a T2*-weighted gradient-echo echo-planar imaging (EPI) sequence and 8-channel array receiving head coil for sensitivity-encoding parallel imaging [41]. The parameters were as follows: echo time (TE) = 35 ms, TR = 2992 ms, field of view (FOV) = 240 × 240 mm, matrix = 96 × 96, flip angle = 90°, number of axial slices = 22, and voxel size = 2.5 × 2.5 × 6.0 mm. Three hundred scans were collected in each session (total time = 2992 ms × 300 = 897,600 ms).

### Image processing

As described in detail previously [42], imaging data were preprocessed and voxel-based statistical analysis performed using SPM8 software (Wellcome Department of Imaging Neuroscience) running on MATLAB (version 8.3.0, Math Works Inc., 2014). The first four volumes were discarded, and the remaining 296 volumes were preprocessed. Preprocessing steps prior to statistical analysis included slice time correction, motion correction, and spatial normalization to a standard template in Montreal Neurological Institute (MNI) space. The slice timing was corrected according to the slice order and the fMRI data realigned and subsequently normalized to the standard MNI template using the T1 SPM template, resulting in voxels of 2 × 2 × 2 mm. All functional images were spatially smoothed using a Gaussian filter kernel (full width at half maximum [FWHM] 8 mm) and the data bandpass filtered (0.008–0.09 Hz). Image artifacts originating from head movement were handled using the ART scrubbing procedure (www.nitrc.org/projects/artifact_detect/), and signal contributions from white brain matter, cerebrospinal fluid, and micro head-movement (six parameters) were discerned from the data. The six realignment parameters were included in the design matrix to correct for signal changes caused by head movement.

Event conditions (omission vs. baseline) were modeled using the standard hemodynamic response function. First level analysis of individual participant images was performed to produce estimates for the contrast of interest (omission vs. tones) according to the general linear model (GLM), and significant signal changes in the contrast were assessed using F-statistics on a voxel-by-voxel basis. The resulting set of voxel values for the contrast constituted statistical parametric mapping (SPM) of the F-statistic. Second level analysis was performed on contrast images in a group random-effects analysis using a one-sample t-test. Two-sample t-tests between groups were performed with age and education as covariates (significance threshold *p* < 0.05, family-wise error [FWE] corrected). Correlation analysis was also performed between BOLD activities and other factors, including the PANSS, ERP, and DTI data. The activated images were superimposed on the standardized T1-weighted images, and the MNI coordinates of the maximum response in the activated regions presented in SPM8 were converted to Talairach coordinates using the algorithm reported by Lacadie et al. [43]. Talairach labels of activated regions were detected using the Talairach client version 2.4.2 (http://www.talairach.org/client.html).

### ROI analysis

Quantitative analysis of the event-related fMRI data was performed using the region of interest (ROI) method centered on our areas of interest in the cingulate cortex and temporal lobe. Estimates of BOLD signal (%) and t-value changes at sound omission were also extracted from select ROIs, including the anterior (S1A Fig) and middle cingulate cortex (S1B Fig), superior and middle temporal gyrus (S1C Fig), and Heschl’s gyrus (HSC; S1D Fig), for each participant using the MarsBar SPM toolbox (MRC Cognition and Brain Sciences Unit, Cambridge, UK; http://marsbar.sourceforge.net) [44].

### Registration and analysis of EEG data

We used a Brain Amp MR-compatible amplifier (Brain Products, Gilching, Germany) to simultaneously record the EEG and fMRI according to previously reported procedures [45–48]. We used a 32-channel EEG cap (Easycap BrainCap-MR 3–0 32Ch) with 30 sintered Ag/Ag Cl ring electrodes placed in accordance with the international 10–10 system. The FCz site (between Fz and Cz) served as the online reference, maintaining a small distance between the recording reference and the “active” electrodes to minimize the risk of amplifier saturation. We placed an electrode on the subject’s back to record the electrocardiogram and another under their left eye to record the electrooculogram. We used custom head coils covered with inner foam padding compatible with the electrodes and cables to decrease head movements by reducing any discomfort or pressure from the electrode cap. We maintained an impedance of <5 kΩ between the electrode and scalp during EEG recording. Data were acquired at a 5000 Hz sampling rate (16 bit, 0.5 mV resolution, 16.38 mV dynamic range) with an online bandpass filter of 0.016–250 Hz.

### EEG artifact correction and validation

We subtracted averaged artifacts [49, 50] using Brain Vision Analyzer software algorithms (BrainProducts, Gilching, Germany) to correct the EEG data for MR gradient and ballistocardiac artifacts as described previously [42]. The EEG data were processed using a bandpass filter of 0.53–70 Hz, down-sampled to 250 Hz, and re-referenced to a common average. We applied additional artifact correction based on independent component analysis (ICA) to exclude repetitive uniform artifacts, such as line noise and eye blinks. All independent components that appeared to correspond to horizontal eye movements, blinks, or components potentially assignable to ballistocardiac and residual gradient artifacts were visually inspected and excluded. We then performed back-projection of the remaining independent components to restore the EEG data to the original voltage and time scales. Finally, we rejected any intervals that included amplitudes > 70 mV.

### ERP analysis

EEG epochs (100 ms pre-stimulus, 412 ms post-stimulus) associated with each stimulus type were excised from the continuous record at the conclusion of the experiment. All single-trial epochs were pre-stimulus baseline-corrected, and artifact-free epochs were segregated by stimulus codes (omission or baseline tone) and averaged for each subject. No significant difference was found in the number of accepted epochs between the conditions, and a group average was calculated across all subjects of each group.

Difference waveforms were constructed by subtracting the tone-associated waveforms from the omission-associated waveforms. Topographic distributions were inspected to verify that the maximum MMN was observed at the Fz or Cz electrodes, where the MMN is typically greatest. Peak MMN amplitudes were detected within latency ranges of 120–185 ms. Analyses were restricted to MMN amplitudes and latencies at Fz and Cz for the purposes of this study, and the MMN amplitudes were measured relative to the pre-stimulus baseline.

### Image acquisition for DTI

All MRI scans for DTI were obtained following image acquisition for fMRI as described previously [41]. Regular structural images, such as T1-weighted spin-echo images, T2-weighted turbo spin-echo images, and fluid-attenuated inversion recovery images, were obtained before the acquisition of diffusion tensor images. DTI was performed utilizing the spin-echo echo-planar technique with the following parameters: echo time = 70 ms, repetition time = 5452 ms, voxel size = 1.75 × 1.75 × 3 mm, number of averages = 2, and contiguous axial slices = 50. Images were obtained with 32-direction diffusion encoding (b = 1000 s/mm^2^ for each direction) and no diffusion encoding (b = 0 s/mm^2^). The scan time was 7 min 17 s.

### DTI analysis

#### Tract-based spatial statistics

As described in detail previously [41], we first carried out voxel-wise statistical analysis as an exploratory analysis using tract-based spatial statistics (TBSS) (S2 Fig), which is available as part of the FMRIB software library (FSL) [51]. Diffusion tensor images were preprocessed by FSL version 4.2.1, including skull stripping and eddy current correction. All subjects’ fractional anisotropy (FA) data were aligned into a common space by applying the non-linear registration tool FNIRT, which uses a b-spline representation of the registration warp field [52]. A mean FA image was provided and thinned to create a mean FA skeleton representing the centers of all tracts common to the group. Group comparisons were performed with Randomise version 2.1 from FSL [53], and both control vs. patient and patient vs. control contrasts were tested with 5000 permutations. We used threshold-free cluster enhancement [54] as implemented within Randomise, which provides the ability to perform cluster-based inferences without setting an arbitrary cluster-forming threshold. Voxel-wise statistical inferences were made on the resulting statistic image, and the significance was set to *p* < 0.05 with correction for FWE.

#### Tract-specific analysis

As our specific interest was the ACC, we performed tract-specific analysis of the 3D diffusion tensor tractograph using dTV II and VOLUME-ONE software (free software from Masutani, http://www.medimg.info.hiroshima-cu.ac.jp/dTV.IISR/dTV.htm) [55] on a Windows PC workstation as described previously [41]. Two ROIs were used to extract the cingulum bundle in accordance with the two-ROI method [55, 56]. The first ROI (‘seed’) was chosen on the coronal plane anterior to the pons (S3A Fig). The second ROI (‘target’) was placed on a coronal slice posterior to the genu of the corpus callosum (S3B Fig). The average FA values and mean diffusivity of the AC included in the seed and target (S3D Fig) were calculated separately for each hemisphere after extracting the cingulum bundle (S3C Fig).

### Statistical analysis

The MMN, ROI, and DTI data were analyzed by analysis of covariance (ANCOVA) with repeated measures using age and education as covariates. Two factors were included for MMN amplitudes and latencies at Fz and Cz: group (patient or control) as a between-subject factor, and electrode (Fz or Cz) as a within-subject factor. Three factors were included for changes in t-values in the ROI analysis of the cingulate and temporal cortex: group as a between-subject factor, and laterality (right or left) and location (anterior or middle cingulate; STG, MTG, or HSC) as within-subject factors. Two factors were included for DTI FA values: group as a between-subject factor, and laterality (right or left) as a within-subject factor. Reduced degrees of freedom (Greenhouse-Geisser) were used when appropriate to counter violations of the sphericity assumption underlying ANCOVA with repeated measures (epsilon values were provided). Changes in the BOLD signal analyzed in ROI analysis using MarsBar were correlated with other fMRI and MMN measures by Pearson’s correlation, and alpha values of 0.05 were considered significant. PASW Statistics software for Windows version 18.0.0 (SPSS Inc., Chicago, IL, USA) was used for all analyses.

## Results

### MMN waveforms

**Fig 2:**
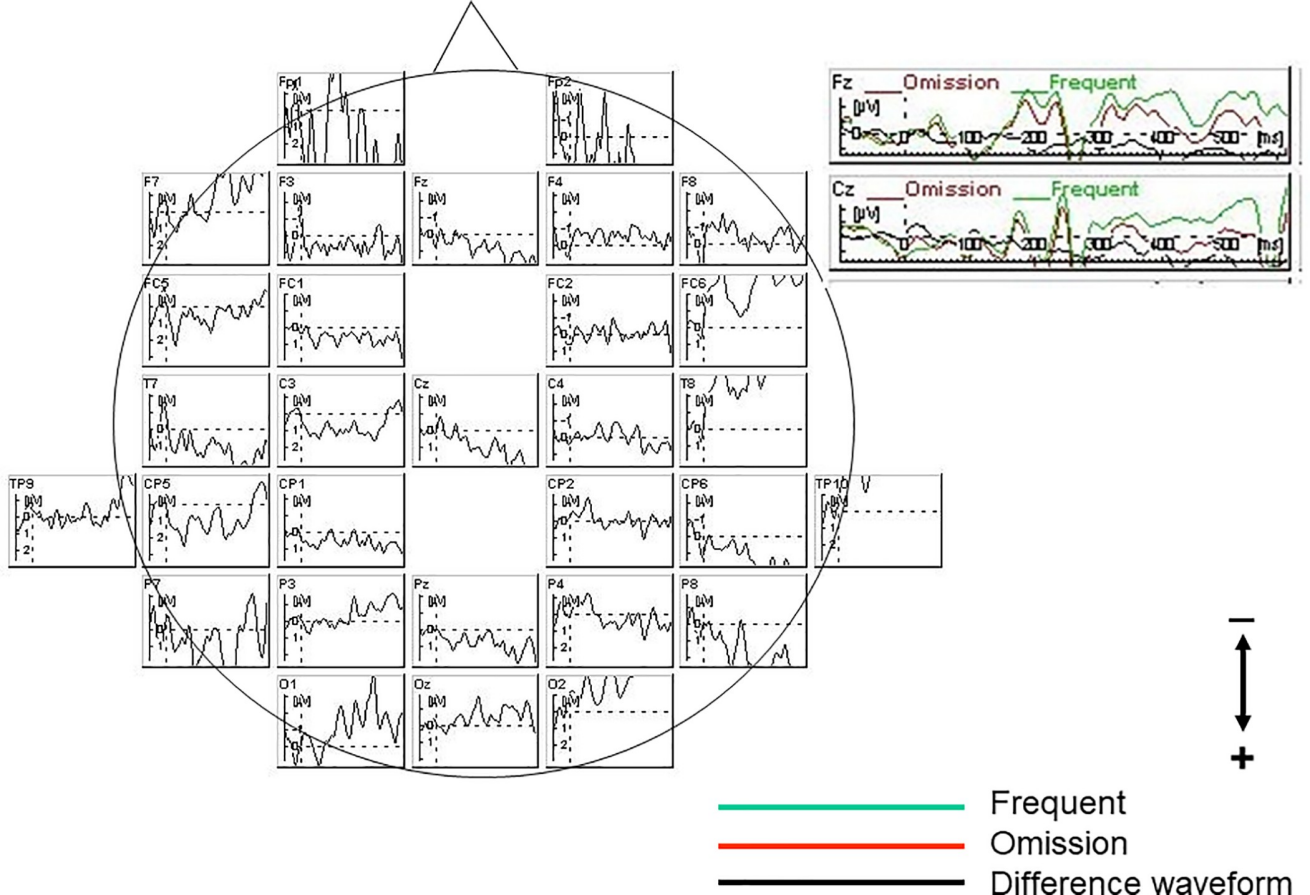
Grand average waveform of controls. Omission events elicited an event-related potential (ERP) component recognized as mismatch negativity (MMN) around latency ranges of 120–185 ms in controls.

**Fig 3:**
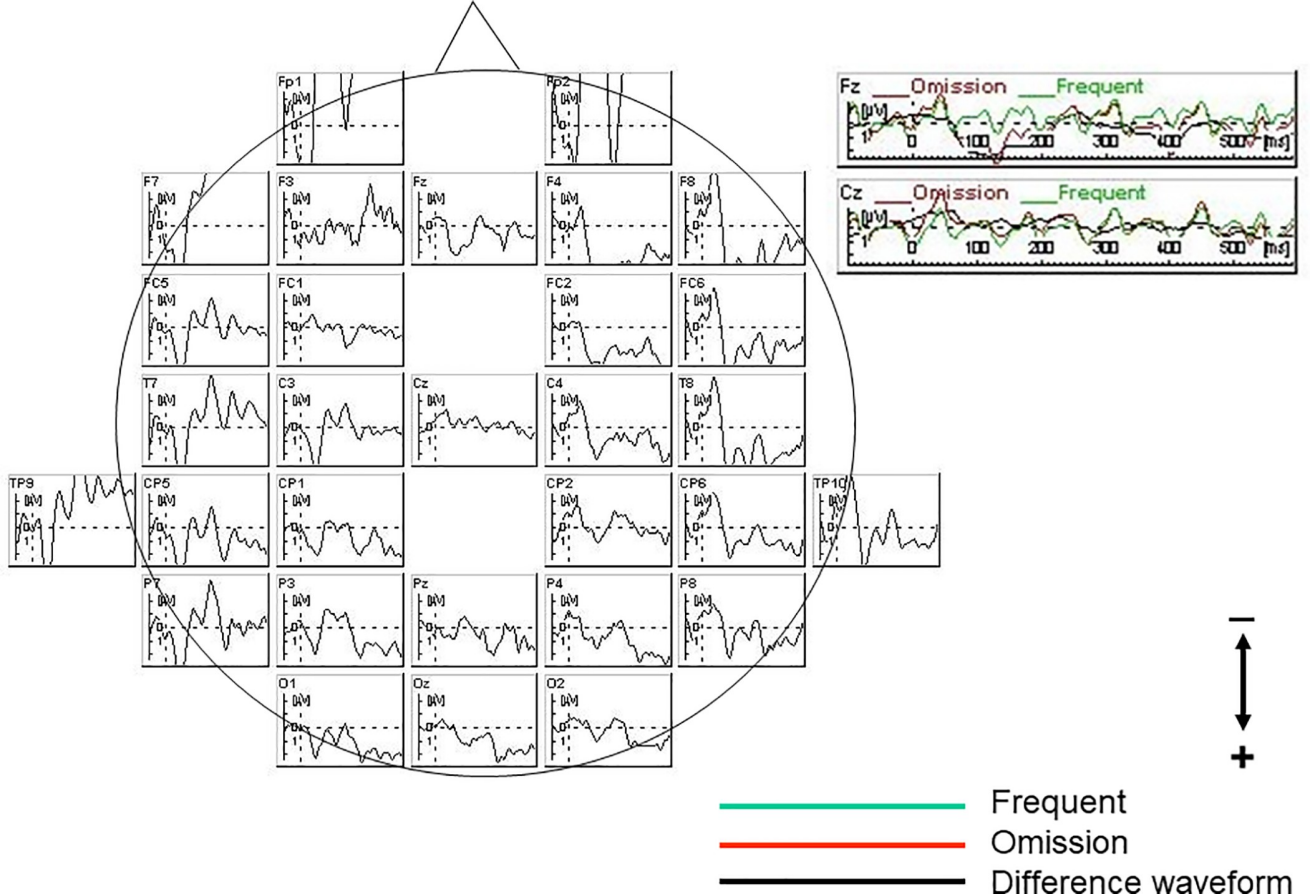
Grand average waveforms of patients. Omission events elicited mismatch negativity (MMN) around latency ranges of 120–185 ms, in patients as well as in controls.

Omission events elicited an ERP component around latency ranges of 120–185 ms in both controls (Fig 2) and patients (Fig 3). ANCOVA with age and education as covariates for MMN amplitudes or latencies revealed no significant main effects or interactions.

### Grand fMRI average in controls

**Fig 4:**
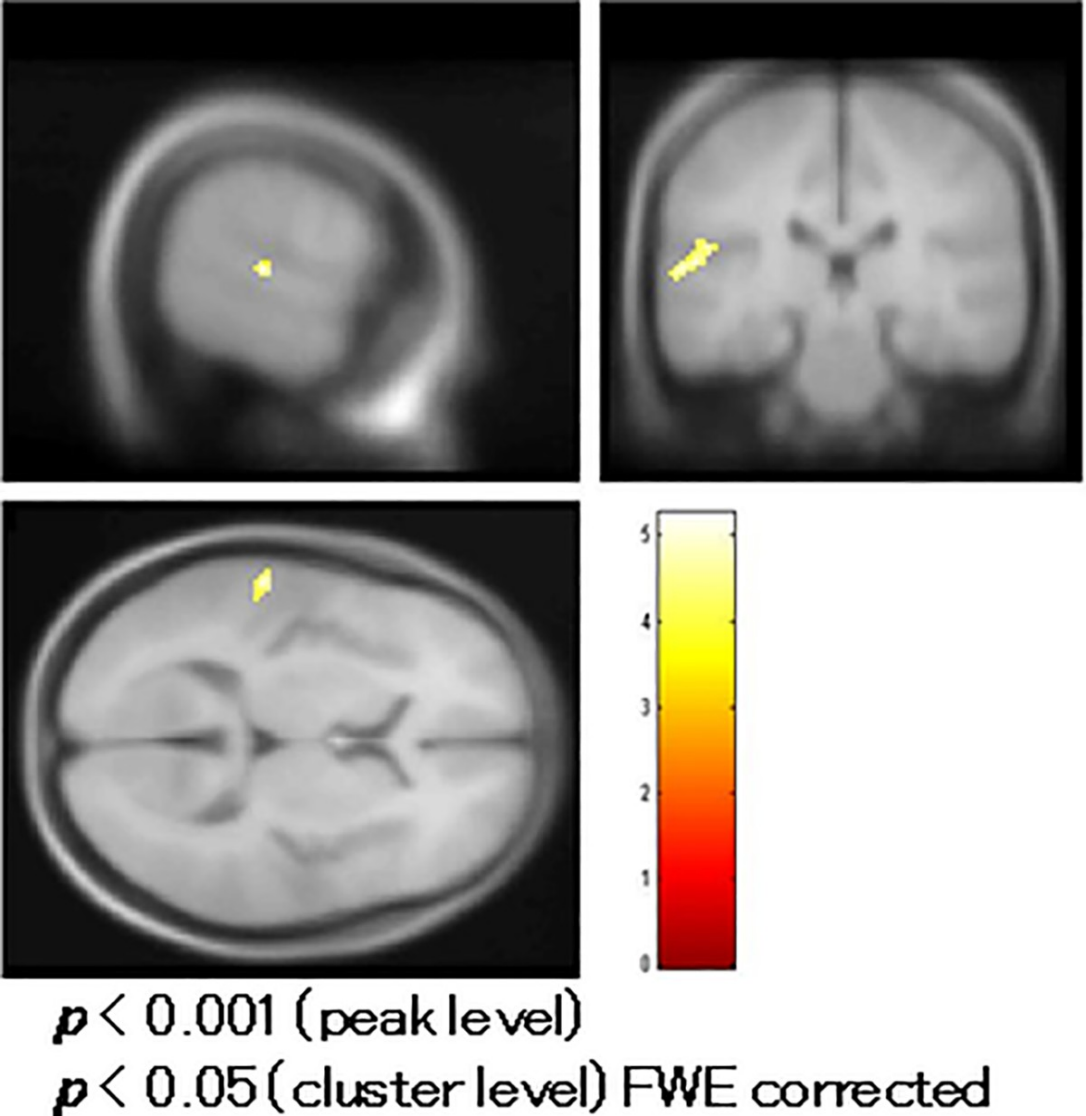
Grand average of functional magnetic resonance imaging (fMRI) in controls. Controls exhibited significant activation responding to omission in the left superior temporal gyrus (Brodmann area/BA 41).

Controls exhibited significant positive activation in response to omission in the left STG (Brodmann area/BA 41) in grand average analysis (Fig 4, Table 2).

**Table 2:** Areas significantly responded to sound omission or correlated to other measures.

Region (BA)	Talairach coordination (X, Y, Z) of peak location	p-value (FWE corrected, cluster level)	t-value (peak level)	Number of significant voxels
**Areas significantly responding to omission of sound in controls (Fig 4).**				
Lt. superior temporal gyrus (BA 41)	(-59.4, -26.8, 8.7)	0.033	5.25	84
**Areas with reduced BOLD activity in the left middle temporal gyrus in patients 8 (Fig 5).**				
Lt. middle temporal gyrus (BA 39)	(-37,6–72.9, 18.4)	0.003	5.35	231
**Areas where BOLD activity significantly correlated positively with MMN amplitude at the Fz site in patients (Fig 6).**				
Rt. superior temporal gyrus (BA 42)	(57.4, -32.5, 10.8)	0.018	6.85	103
**Areas where BOLD activity significantly correlated positively with PANSS scores of positive symptoms (Fig 7)**				
Rt. parahippocampal gyrus (BA 19)	(41.6, -42.6, 2.1)	0.000	8.32	392
**Areas where BOLD activity significantly correlated positively with PANSS scores of hostility and Positive symptoms according to Bell’s classification (Fig 8)**				
**correlated with hostility**				
Rt. Insula (BA 13)	(33.7, -41.8, 18.7)	0.003	6.87	146
**correlated with positive symptoms**				
Rt. parahippocampal gyrus (BA19)	(39.6, -40.7, 2.0)	0.000	9.19	486
**Areas where BOLD activity significantly correlated positively with BOLD signal change in the bilateral Heschl’s Gyri. (Figs 10 and 11).**				
**correlated Lt. Heschl gyrus**				
Lt. insula (BA 13)	(43.6, -15.2, 6.3)	0.000	12.20	1107
Lt. postcentral gyrus (BA43)	(63.4, -10.6, 20.8)	0.000	21.46	218
Lt. inferior parietal lobule (BA 40)	(-55.4, -25.5, 34.4)	0.020	6.75	97
Rt insula	(—4.0 -40.0, 14.9)	0032	5.57	88
**correlated with Rt. Heschl gyrus**				
Rt. insula (BA 13)	**(**43.6, 3.5, -6.9)	0.018	6.13	105
Lt. posterior cingulate(BA29)	(-2.0, -42.0, 15.0)	0.008	7.96	123
Lt. middle frontal gyrus (BA 9)	(-29.7, 30.3, 22.4)	0.005	5.60	134

BA: Brodmann area

FWE: family-wise error rate

### Comparison of fMRI between patients and controls

**Fig 5:**
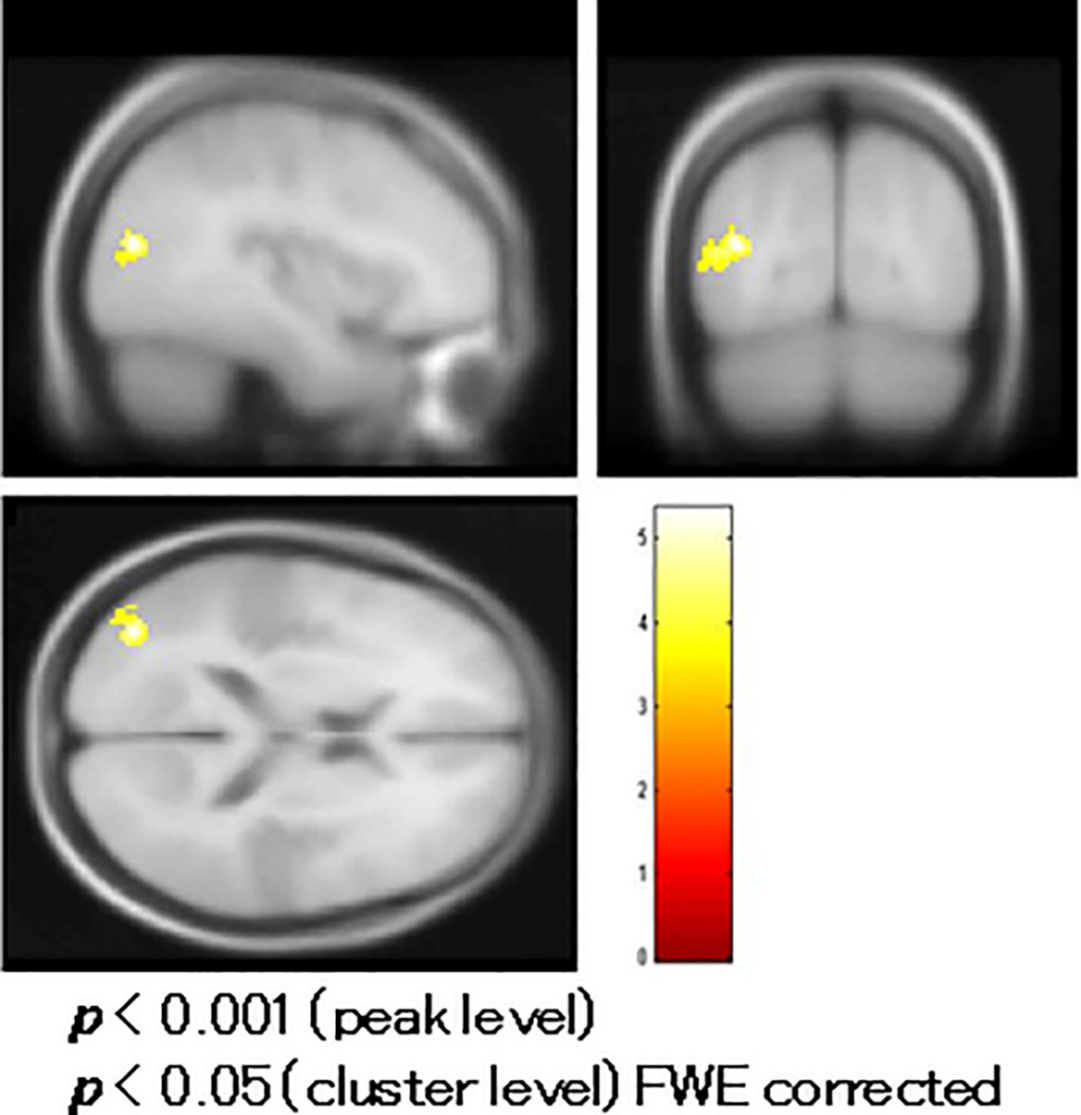
Comparison of functional magnetic resonance imaging (fMRI) between patients and controls. Patients with schizophrenia had reduced blood oxygenation level-dependent (BOLD) activity in the left middle temporal gyrus (Brodmann area/BA 39) compared to controls in a t-test with age and education as covariates.

Schizophrenic patients had reduced BOLD activity in the left middle temporal gyrus (MTG) (BA 39) compared to controls in a t-test with age and education as covariates (Fig 5, Table 2).

### Correlation analysis of fMRI in patients

**Fig 6:**
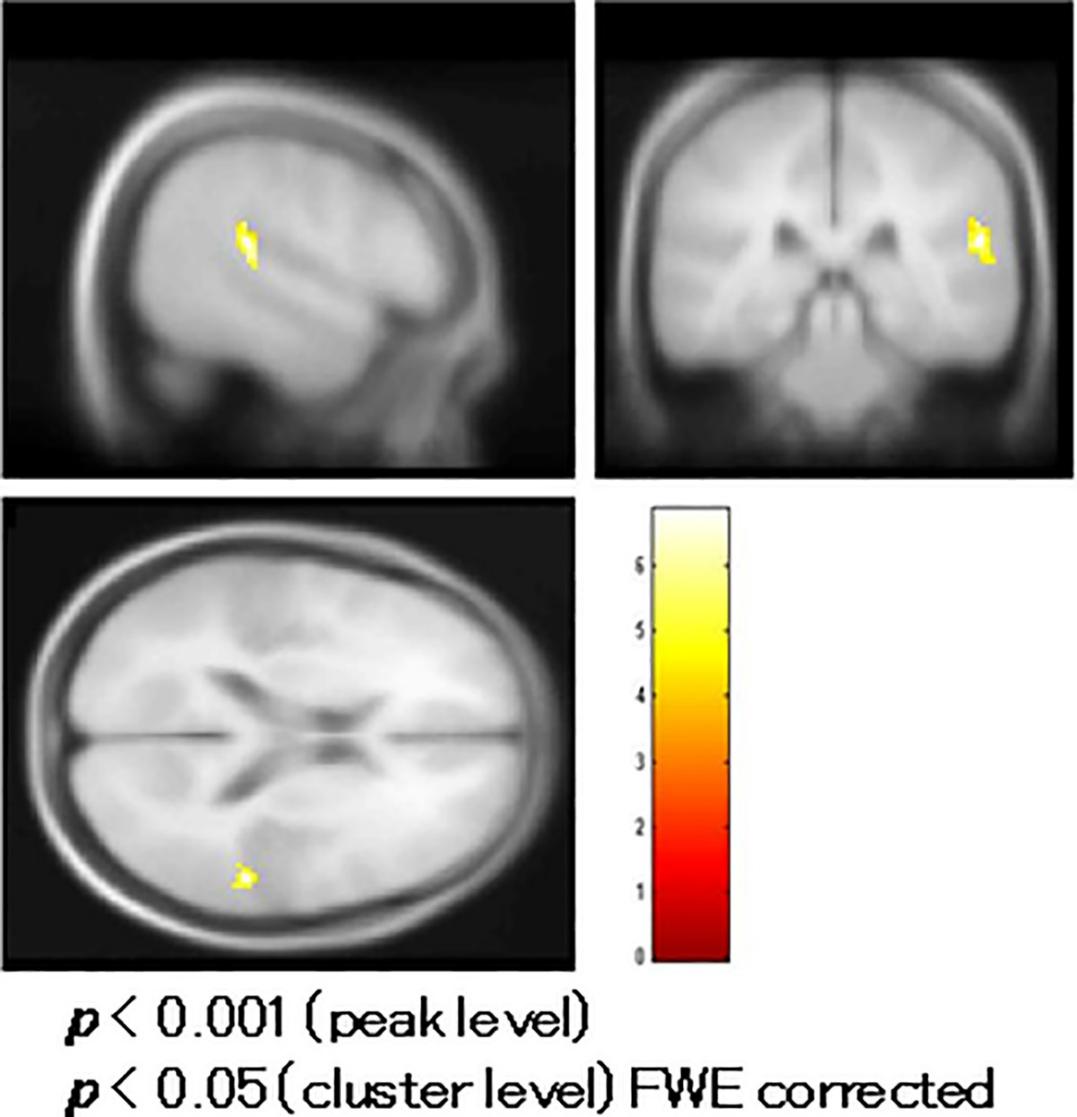
Correlation of functional magnetic resonance imaging (fMRI) with mismatch negativity (MMN) in patients. Blood oxygenation level-dependent (BOLD) activity in the right superior temporal gyrus significantly correlated with MMN amplitudes at the Fz site.

**Fig 7:**
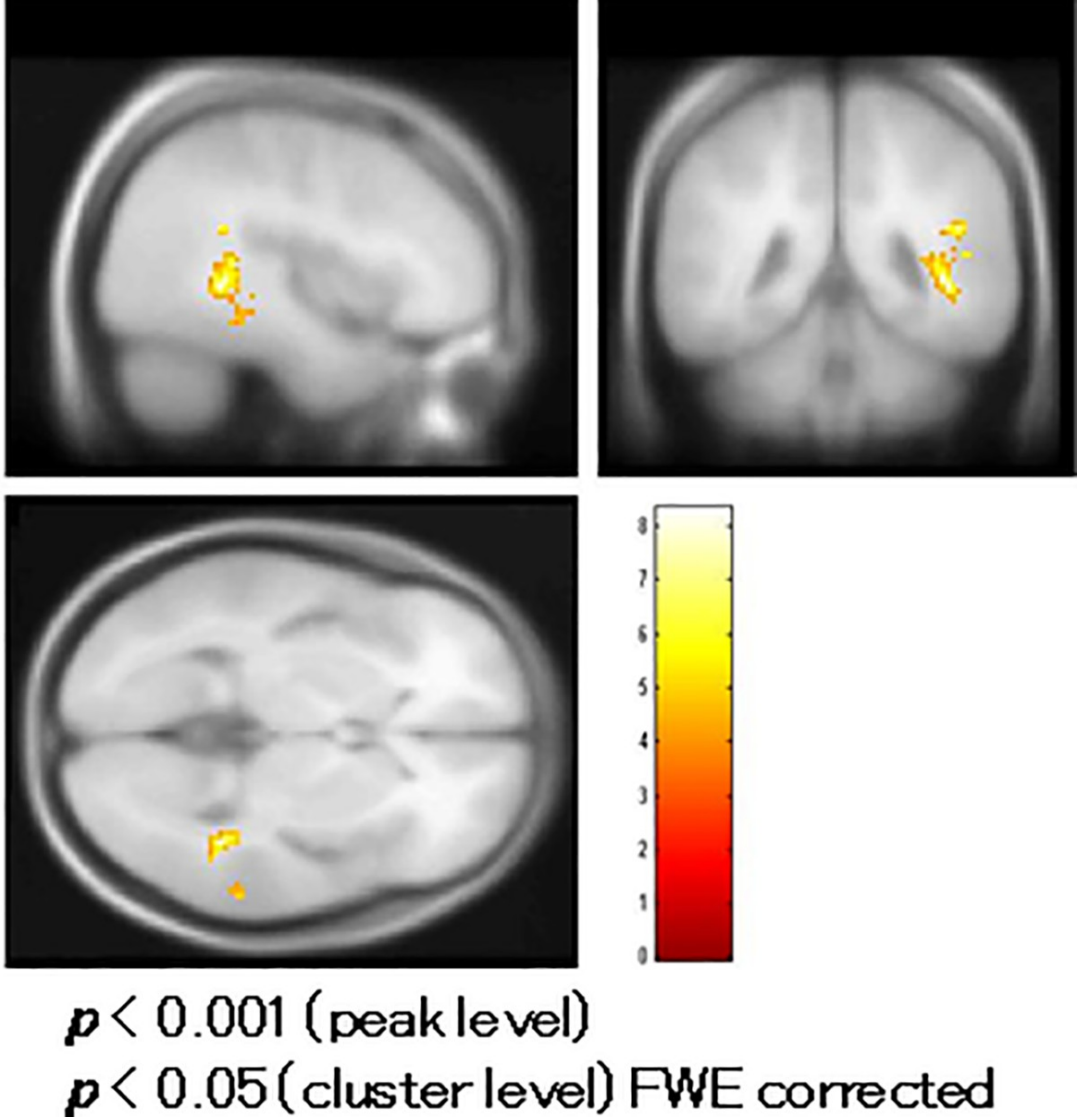
Correlation of functional magnetic resonance imaging (fMRI) with the PANSS in patients. Blood oxygenation level-dependent (BOLD) activity in the right parahippocampal gyrus correlated with the Positive and Negative Syndrome Scale (PANSS) positive symptom subscale scores.

**Fig 8:**
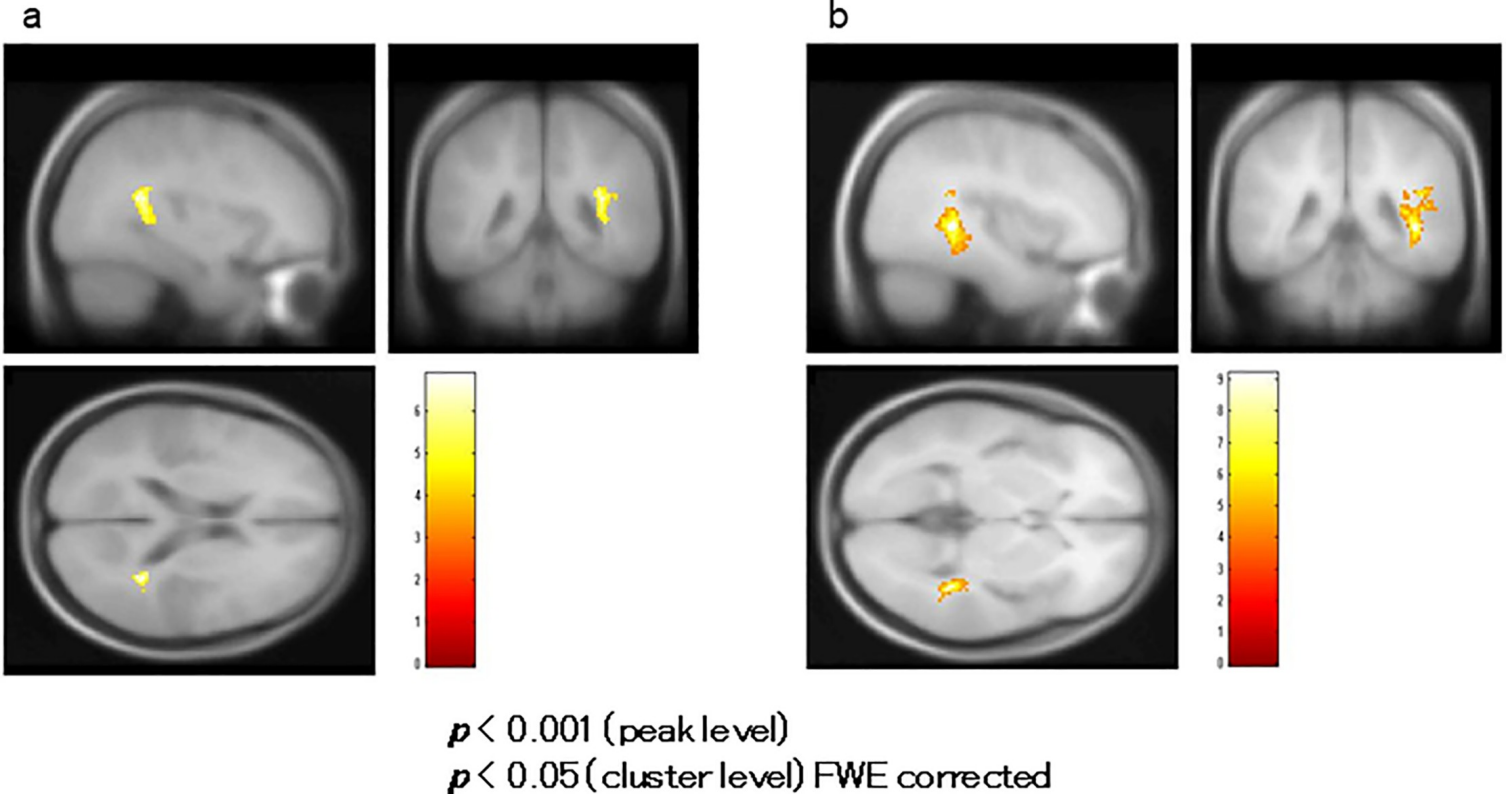
Correlation of functional magnetic resonance imaging (fMRI) with Bell’s PANSS classification. (a) Blood oxygenation level-dependent (BOLD) activity in the right insula and (b) right parahippocampal gyrus significantly correlated with the Positive and Negative Syndrome Scale (PANSS) hostility and positive symptom subtotal scores, respectively, as classified by Bell’s classification.

BOLD activity of the right superior temporal gyrus significantly correlated positively with MMN amplitudes at the Fz site in the correlation analysis (Fig 6, Table 2). The BOLD activity of the right parahippocampal gyrus correlated positively with PANSS positive symptom subtotal scores (Fig 7, Table 2). The BOLD activity of the right insula (Fig 8A, Table 2) and right parahippocampal gyrus (Fig 8B, Table 2) significantly correlated positively with hostility and positive symptom subtotal PANSS scores as defined by Bell’s classification.

### ROI analysis

**Fig 9:**
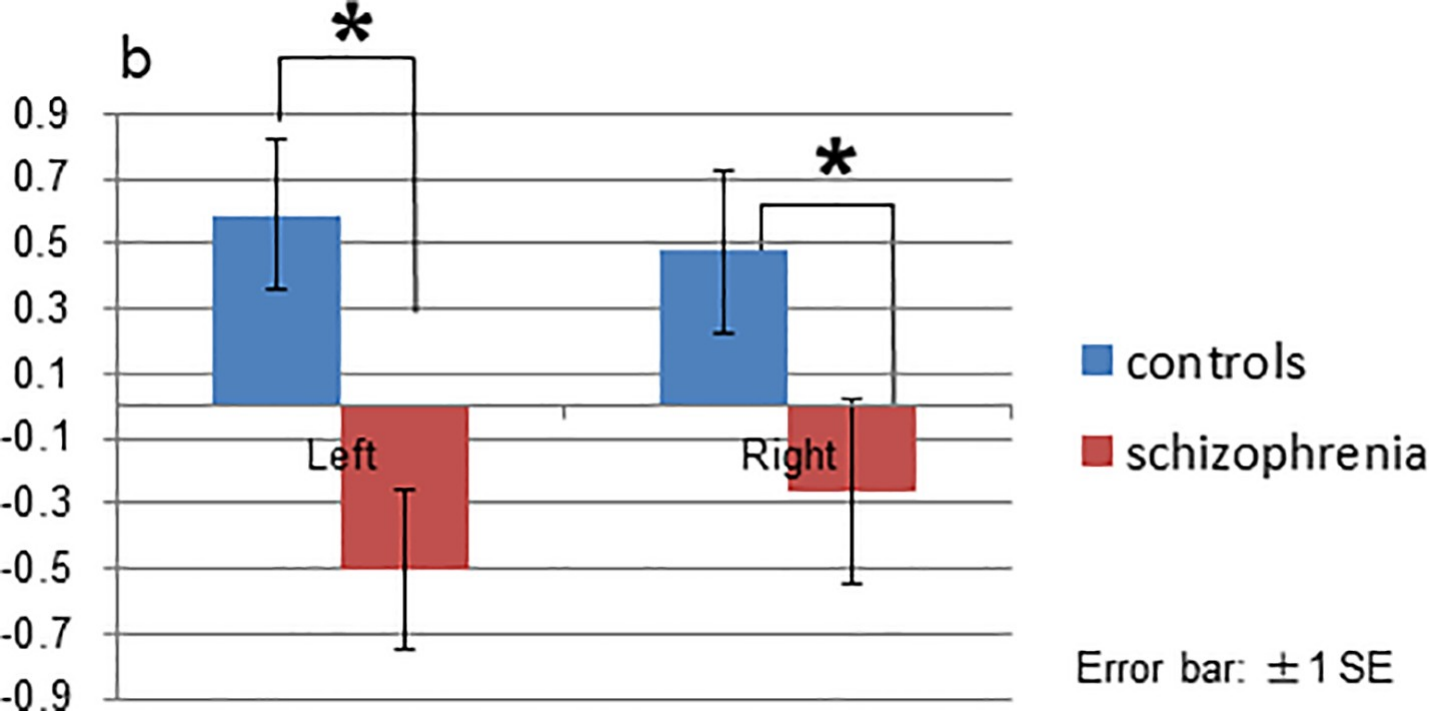
ROI (region of interest) analysis of the temporal gyrus in the middle temporal gyrus. Patients demonstrated reduced signal change (t-values) in the bilateral middle temporal gyrus compared to controls.

ANCOVA of the ROI analysis revealed no significant main effects or interactions in the cingulate cortex (S4A and S4B Fig). The group effect [F(1, 23) = 5.239, *p* = 0.032] and interaction between group and location [F(2, 46) = 3.675, *p* = 0.033] were significant in the temporal cortex. Post-hoc tests with Bonfferoni correction indicated that patients had a reduced signal change (t-values) in the bilateral middle temporal gyrus compared to controls (left *p* = 0.002, right *p* = 0.040; Fig 9), though no significant differences were found in the STG (S5A Fig) or HSC (S5B Fig).

In patients, changes in the BOLD signal (t-values) in the left HSC in ROI analysis significantly correlated positively with MMN amplitudes at the Fz site (Pearson’s correlation: R = 0.583, *p* = 0.045; S6 Fig).

**Fig 10:**
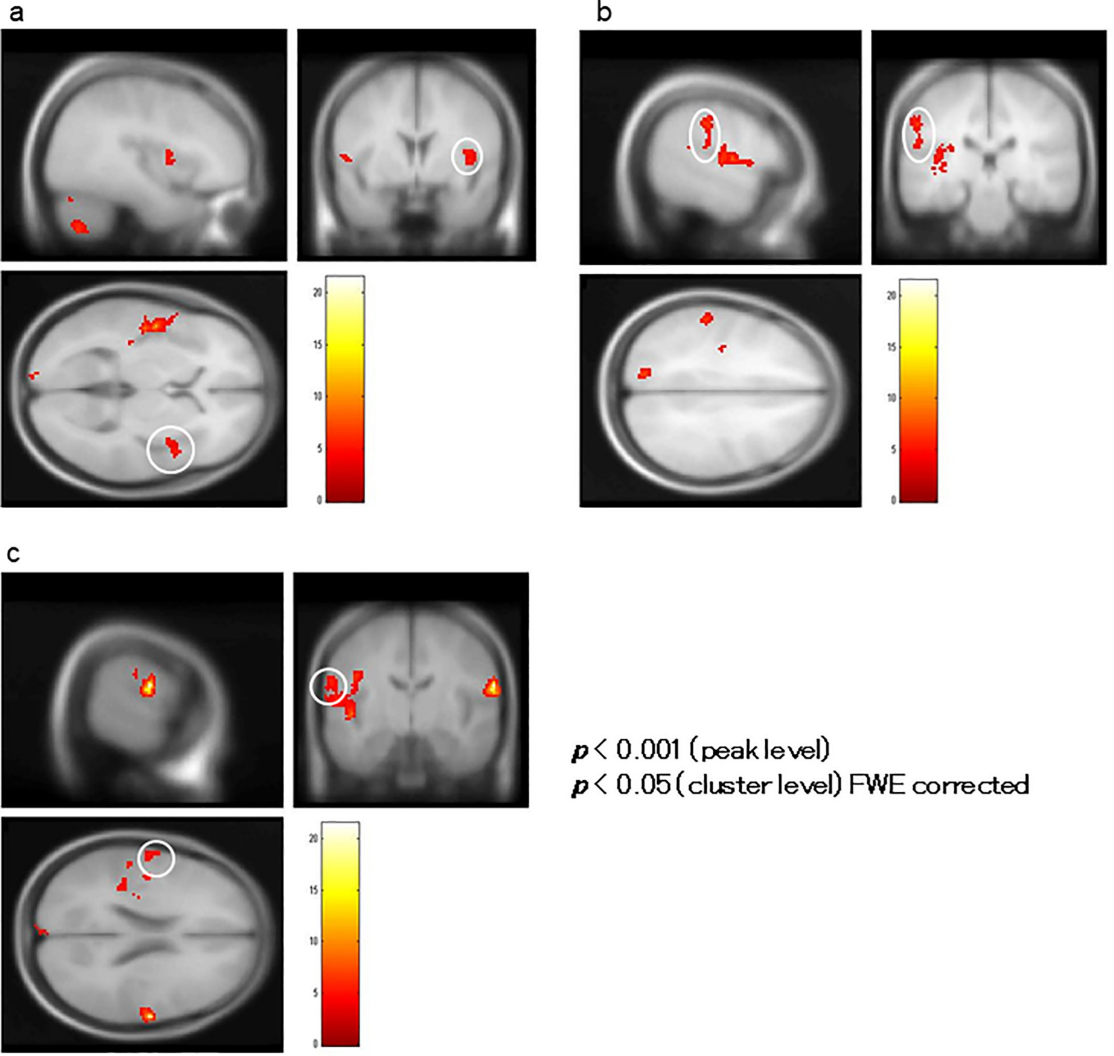
Correlation analysis of BOLD signal changes in the left Heschl’s Gyrus (HSC). In a correlation analysis using blood oxygenation level-dependent (BOLD) signal change (%) in the HSC as a covariate in ROI analysis, BOLD activity in the left insula encompassing the superior temporal gyrus (not shown), right insula (indicated by the white circle in (a)), right postcentral gyrus (indicated by the white circle in (b)), and left inferior parietal lobule (indicated by the white circle in (c)) significantly correlated with BOLD signal changes in the left HSC.

**Fig 11:**
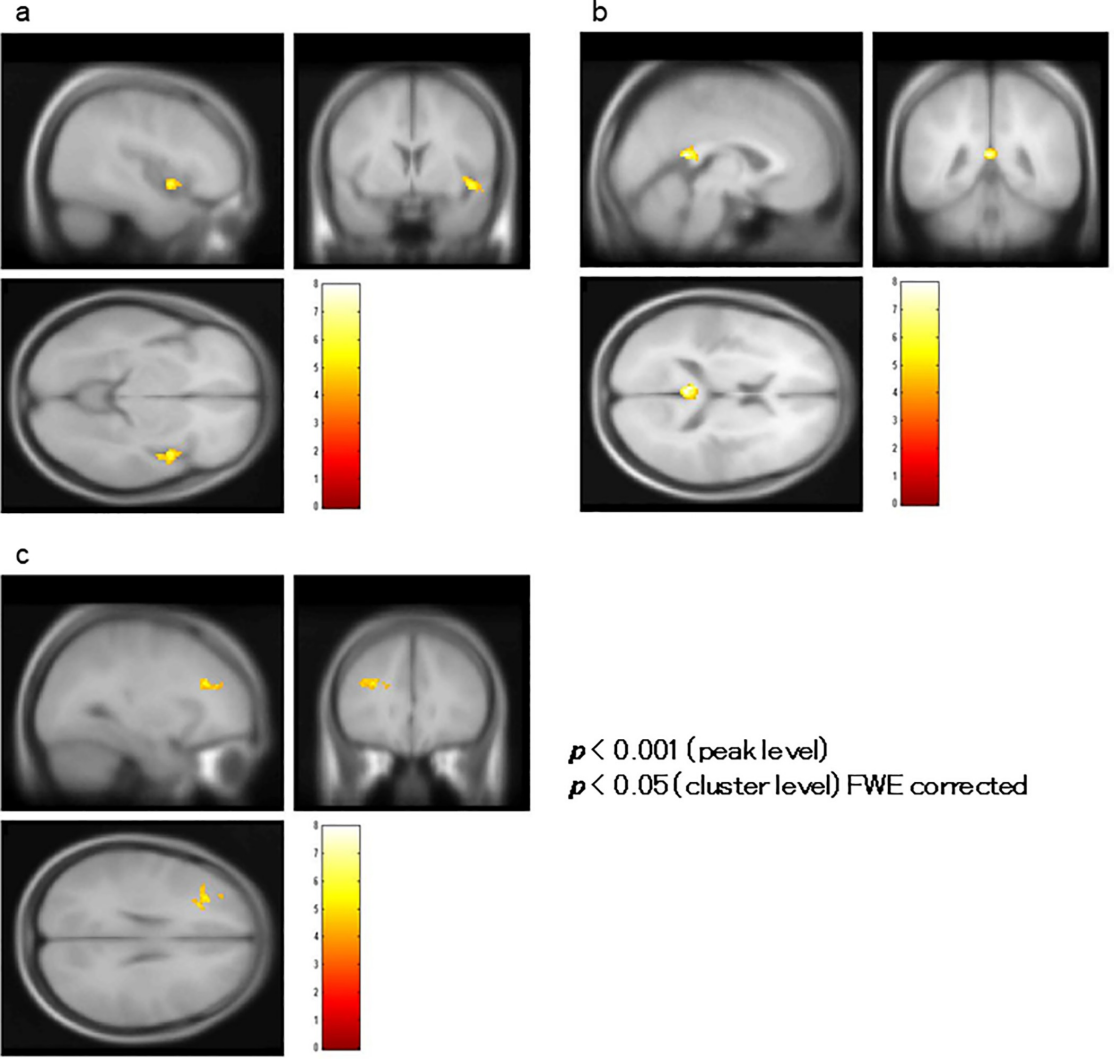
Correlation analysis of BOLD signal changes in the right Heschl’s Gyrus (HSC). (a) Blood oxygenation level-dependent (BOLD) activity in the right insula, (b) left posterior cingulate, and (c) left middle frontal gyrus significantly correlated with that of the right HSC of patients.

A correlation analysis using BOLD signal change (%) in the bilateral HSC as the covariate showed that BOLD activity in the left insula (BA 13) encompassing the STG (BA 22), right insula (Fig 10A), right postcentral gyrus (BA 43; Fig 10B), and left inferior parietal lobule (BA 40; Fig 10C) significantly correlated positively with changes in the BOLD signal in the left HSC, and BOLD activity in the right insula (BA 13; Fig 11A), left posterior cingulate (BA 29; Fig 11B), and left middle frontal gyrus (BA 9; Fig 11C) significantly correlated positively with the activity in the right HSC (Table 2).

### DTI

**Fig 12:**
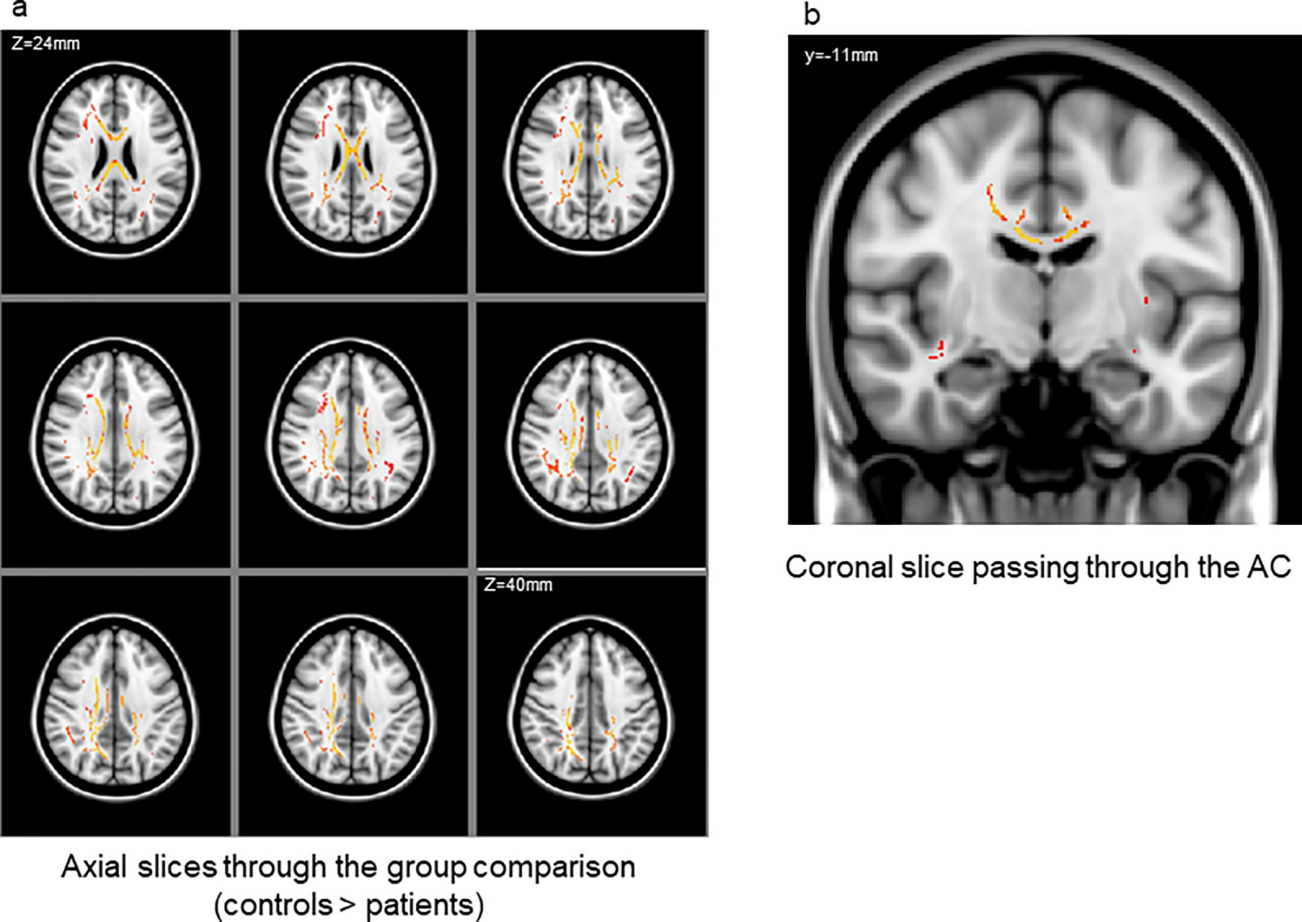
Regions of significant fractional anisotropy (FA) reduction in patients with schizophrenia relative to controls (red-yellow, *p* < 0.05, corrected for family-wise error). (a) Axial slices from Z = 24 to Z = 38 mm and (b) a coronal slice show the bilateral anterior cingulum region along with the right posterior cingulum. The left-right orientation is in accordance with the radiological convention.

**Fig 13:**
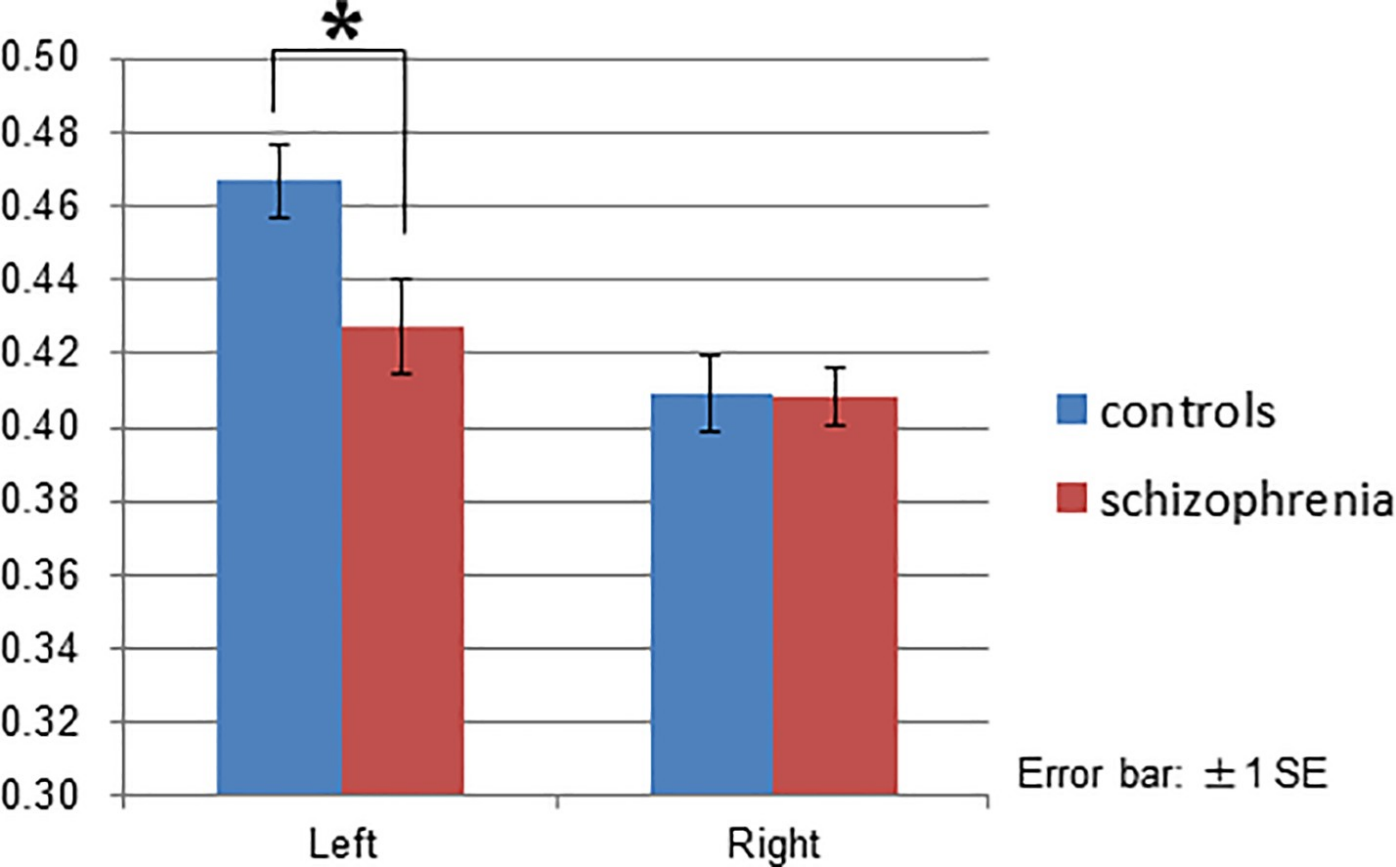
Fractional anisotropy (FA) values in the anterior cingulate gyrus. Patients had reduced FA values in the left anterior cingulate gyrus.

TBSS revealed a cluster of significant FA reductions in patients compared to controls. This cluster included the bilateral anterior cingulate, right posterior cingulate, deep white matter in the frontal, temporal, and parietal lobes, a large portion of the corpus callosum, and the corona radiata (Fig 12A and 12B). Although ANCOVA for FA values of the ACC revealed no significant main effects or interactions, post-hoc tests with Bonfferoni correction indicated that patients had reduced FA values in the left anterior cingulate gyrus (*p* = 0.49; Fig 13). FA values in the left ACC significantly correlated positively with BOLD signal changes (t-values) in the right STG (S7 Fig, Pearson’s correlation, R = -0.583, *p* = 0.045), and FA values in the right anterior cingulate gyrus significantly correlated negatively with total PANSS scores (S8A Fig; Pearson’s correlation, R = -0.632, *p* = 0.027) and Emotional Discomfort subtotal score as defined by Bell’s classification (R = -0.764, *p* = 0.004; S8B Fig).

## Discussion

The left STG was activated by the omission deviant in the controls in accordance with previous reports identifying the MMN generator in that locus [1, 9, 14, 15, 57]. Patients with schizophrenia exhibited reduced BOLD activity in the left MTG relative to controls, which is also in line with previous reports of attenuated MMN in schizophrenia [8, 58–62]. The significant correlation between BOLD activity in the right STG and MMN amplitudes suggests that the phenomenon seen on fMRI may be a counterpart to the MMN component recorded on the EEG.

BOLD activity in the right insula and right parahippocampal gyrus significantly correlated with PANSS positive symptoms and hostility subscores, and we found a correlation between BOLD activation in the HSC and the limbic system, including the insula. The association between BOLD activation in the right insular/parahippocampal gyrus and PANSS hostility/positive symptom scores is consistent with previous reports suggesting that the anterior insular cortex is involved in experiencing negative emotions [63, 64], especially disgust [65, 66].

Chen et al. used a passive oddball paradigm consisting of emotionally meaningless syllables spoken with neutral, happy, or disgusted prosodies, along with acoustically matched simple and complex tones, and demonstrated that, relative to happy syllables, disgusted syllables elicited stronger MMN-related cortical activities measured by magnetoencephalography (MMNm) in the right anterior insular cortex, precentral gyrus, left posterior insular cortex, supramarginal cortex, transverse temporal cortex, and upper bank of the superior temporal cortex. This finding supports a role of the anterior insular cortex in pre-attentive processing of emotional salience [67]. Using an MMN/field paradigm, Ford et al. reported significantly reduced right hemisphere fronto-temporal and insular cortex activation in a “high social disorganization” group sharing autistic and schizotypal phenotypes, and indicated that right fronto-temporal and insular cortex-related auditory change processing may be associated with psychosocial functioning [68]. The insular cortex not only relays information to and from somatosensory, auditory, and visual areas and the amygdala, which has been suggested to be involved in emotion and fear processing [64], but is among the secondary auditory cortices, with connections to the STG and dorsal superior temporal sulcus [69]. The posterior insular cortex is part of the extended auditory cortex and preferentially responds to vocal commutation [70]. The detection of auditory changes in the primary auditory cortex triggers a pre-attentive reorienting response in the right frontal lobe, with feedback to the auditory sensory regions through connections between the IFG and STG [17]. Changes in the structure of the insular cortex have been identified in patients with schizophrenia and autism [71]. Connections between these regions may be disrupted in schizophrenia, disrupting feedback pathways from the IFG to the STG, suggesting a deficit in the connection between the auditory processing regions and the IFG [68]. These previous literatures suggesting a key role of the fronto-temporal-limbic cortical networks in MMN generation may support our findings that BOLD activation of Heschl’s gyri correlated with that of the limbic system, including the insula.

The association between BOLD activity in the right parahippocampal gyrus and PANSS scores may be relevant to previous findings. In bipolar disorder, disturbances in the hippocampal glutamate/NMDA system are potentially related to a lack of tightly regulated hippocampal NMDA functioning [72, 73]. Although mismatch responses are attenuated in patients with schizophrenia, a failure of the inhibitory mechanism in the limbic system or increased distractibility when detecting mismatch stimuli may contribute to clinical manifestations.

FA values in the left ACC significantly correlated with BOLD signal changes in the right STG, and FA values in the right ACC significantly correlated with PANSS scores. These results are in line with previous studies that suggested the ACC is a source of MMN [10–12]. Takahashi et al. examined the neural sources of MMN and P3a components in a large cohort of patients with schizophrenia using exact low resolution electromagnetic tomography analysis (eLORETA) and comparing them to non-psychiatric control subjects. Reduced activation of discrete medial frontal brain regions, including the anterior–posterior cingulate and medial frontal gyri, were associated with MMN deficits, whereas deficits in P3a were associated with reduced activation of attentional networks (i.e., frontal, temporal, and parietal regions) [74]. Their results support the hypothesis that a distributed neural architecture supporting initial auditory sensory discrimination, such as MMN processing, is similar in patients with schizophrenia, but impairments in the medial frontal region devolve into generalized neurocognitive deficits [75]. Among the core neurophysiological features of schizophrenia is abnormal activation of medial frontal areas, including the cingulate cortex and medial frontal gyrus, accounting for several distinct cognitive deficits [76]. The associations between ACC structure and the mismatched BOLD response in the right STG or total PANSS scores observed in this study are consistent with hierarchical information processing models of cognitive deficits that have been proposed for patients with schizophrenia [77]. In schizophrenia, impaired automatic stimulus discrimination may contribute to the higher-order cognitive and psychosocial deficits [74], and changes in ACC structure may be part of the neural basis of MMN changes and disruption of the limbic-cortical structural networks. Although causal inferences should be considered with caution given the limitations, the deficits of the feedback/feed-forward connection between the prefrontal cortex and STG modulated by the ACC and insula may specifically contribute to impaired MMN generation and clinical manifestations in schizophrenia.

This study has several limitations. First, these findings are underpowered due to the relatively small sample size with non-matched groups regarding age and education. The small patient population may have contributed to the lack of significant differences in the MMN amplitudes or latencies, and additional studies with larger samples are needed to elucidate the clinical implications of the present findings. There is also a need for stricter noise reduction of raw EEG data by reprocessing the data, as insufficient artifact correction may have contributed to the negative findings related to MMN amplitude or latency. Finally, DTI ROIs are limited to the ACC. A broader range of ROIs including the limbic system, STG, and IFG should help elucidate the impaired connection between the STG, ACC, and insula in schizophrenia. Further analysis of fMRI, EEG, and DTI data in functional brain networks is necessary for improved understanding of the cognitive function pathophysiology seen in schizophrenia.

## Conclusion

This is the first study to examine MMN using simultaneous fMRI, EEG, and DTI recording in patients with schizophrenia to determine the implications of abnormalities in the ACC, STG, and limbic system, including the insula and parahippocampal gyrus, and understand their dysfunctions as reflected by MMN. The ACC structural changes occurring in schizophrenia may be part of the neural basis underlying MMN deficits, and deficits in the feedback/feed-forward connection between the prefrontal cortex and STG modulated by the ACC and insula may specifically contribute to impaired MMN generation and clinical manifestations.

## Supporting information

S1 Fig ROIs (region of interest) defined by MarsBar software. (a) anterior and (b) middle cingulate cortex, (c) superior and middle temporal gyrus, and (d) Heschl’s gyrus.
(TIF)

S2 Fig Diffusion tensor imaging (DTI) data processing by tract-based spatial statistics (TBSS). Fractional anisotropy (FA) data from all subjects were aligned into a common space. A mean FA image was provided and thinned to create a mean FA skeleton representing the centers of all tracts common to the group.
(TIF)

S3 Fig Delineation of the cingulum bundles. (A, B) Sagittal (left) and coronal (right) slices of the color map. The ‘seed’ (blue) and ‘target’ (pink) ROIs (region of interest) were defined to delineate the cingulum bundle. (C) 3D view of the extracted cingulum bundle. The tracts between the seed and target were voxelized. (D) Fractional anisotropy (FA) values in the voxels were calculated.
(TIF)

S4 Fig ROI (region of interest) analysis in the cingulate cortex. (a) No significant differences in Blood oxygenation level-dependent (BOLD) signal changes (t-values) were found in the bilateral anterior cingulate cortex (ACC) or (b) posterior cingulate cortex.
(TIF)

S5 Fig ROI (region of interest) analysis of the temporal gyrus in the superior temporal gyrus and Heschl’s gyrus. No significant differences were found between patients and controls in the superior temporal gyrus (a) and Heschl’s gyrus (b). Blood oxygenation level-dependent (BOLD) signal changes (t-values) in the left HSC in ROI analysis significantly correlated with MMN amplitudes at the Fz site in patients.
(TIF)

S6 Fig Correlation between BOLD signal changes in the left Heschl’s gyrus (HSC) and mismatch negativity (MMN). Blood oxygenation level-dependent (BOLD) signal changes (t-values) in the left HSC in ROI analysis significantly correlated with MMN amplitudes at the Fz site in patients.
(TIF)

S7 Fig Correlation between BOLD signal changes in the right superior temporal gyrus (STG) and fractional anisotropy (FA) values in the left anterior cingulate cortex (ACC). FA values in the left ACC significantly correlated with blood oxygenation level-dependent (BOLD) signal changes in the right STG of patients.
(TIF)

S8 Fig Correlation between PANNS scores and fractional anisotropy (FA) values in the right anterior cingulate cortex (ACC). (a) FA values in the right ACC significantly correlated with the Positive and Negative Syndrome Scale (PANSS) total scores and (b) Emotional Discomfort subtotal score as classified by Bell’s classification.
(TIF)
