# Supplementary figures and images for: Simultaneous fMRI-EEG-DTI recording of MMN in patients with schizophrenia
Source: PLoS One. 2019 May 9;14(5):e0215023. doi: 10.1371/journal.pone.0215023 (PMC6508624; doi:10.1371/journal.pone.0215023)

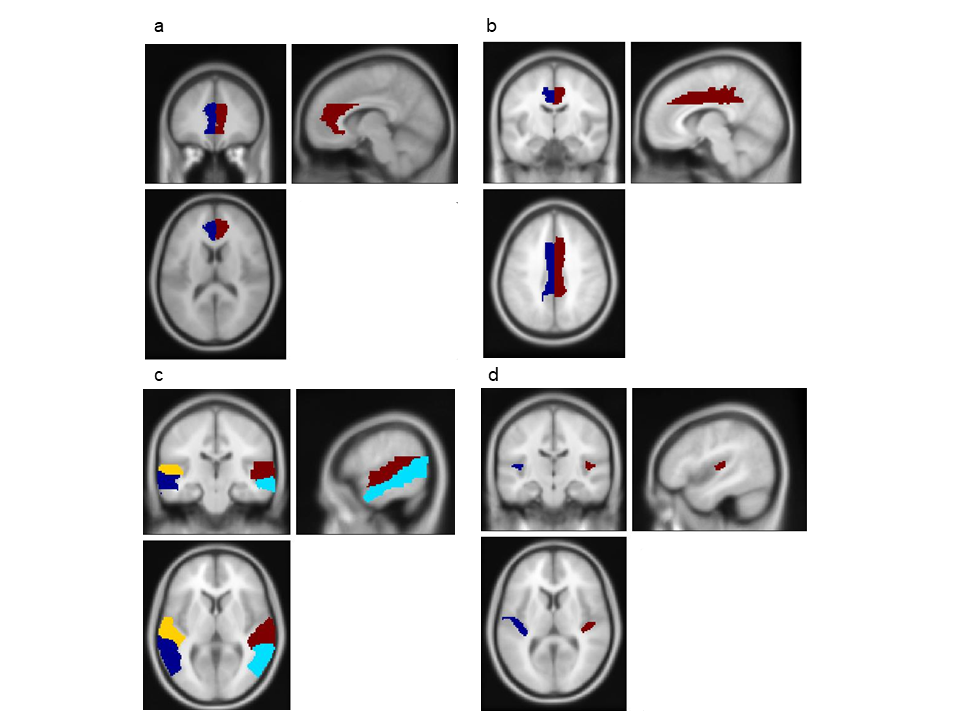

Supplement: S1 Fig — (a) anterior and (b) middle cingulate cortex, (c) superior and middle temporal gyrus, and (d) Heschl’s gyrus. (TIF) [file pone.0215023.s001.TIF]

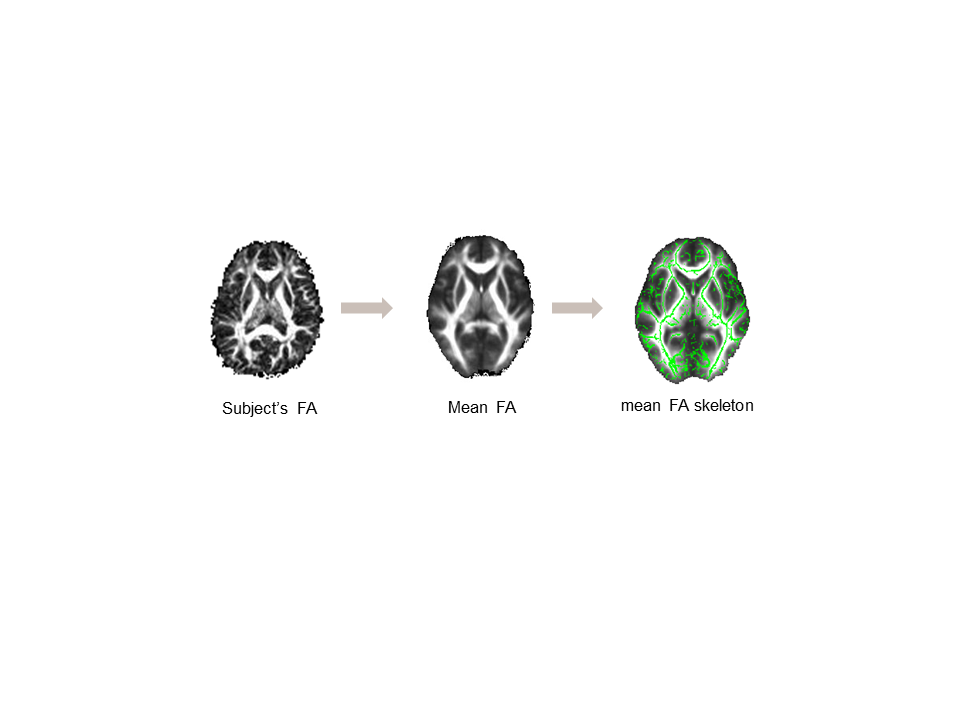

Supplement: S2 Fig — Fractional anisotropy (FA) data from all subjects were aligned into a common space. A mean FA image was provided and thinned to create a mean FA skeleton representing the centers of all tracts common to the group. (TIF) [file pone.0215023.s002.TIF]

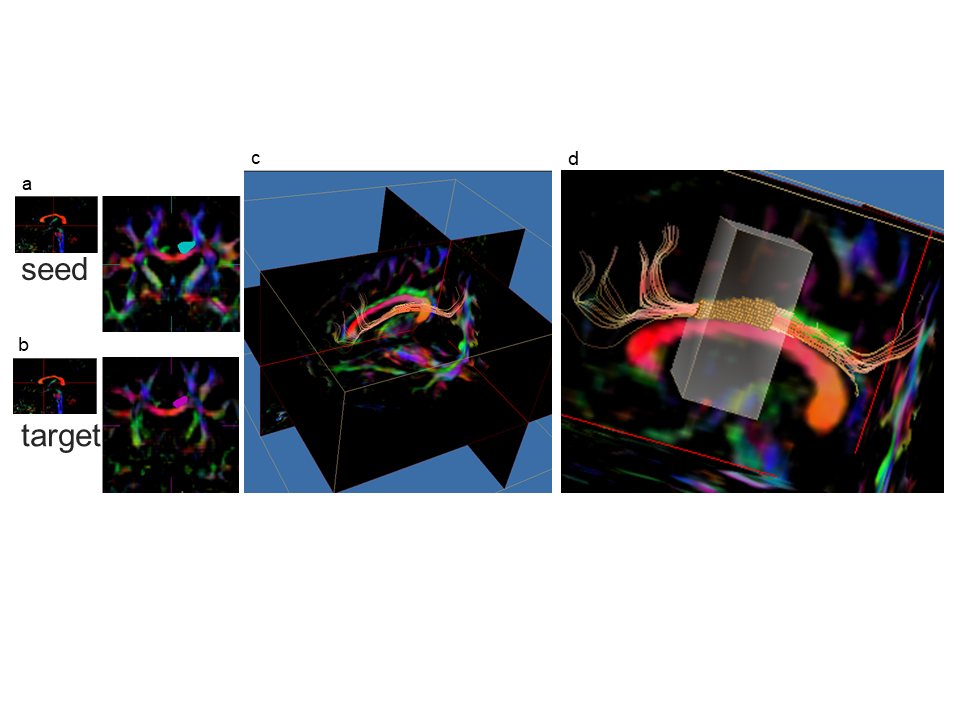

Supplement: S3 Fig — (A, B) Sagittal (left) and coronal (right) slices of the color map. The ‘seed’ (blue) and ‘target’ (pink) ROIs (region of interest) were defined to delineate the cingulum bundle. (C) 3D view of the extracted cingulum bundle. The tracts between the seed and target were voxelized. (D) Fractional anisotropy (FA) values in the voxels were calculated. (TIF) [file pone.0215023.s003.TIF]

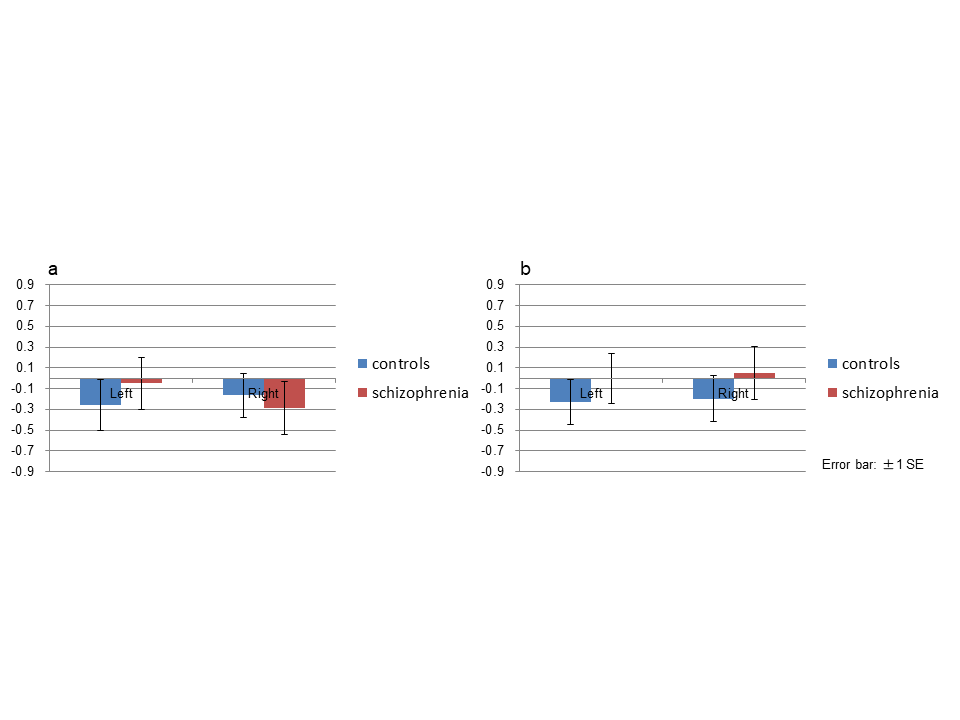

Supplement: S4 Fig — (a) No significant differences in Blood oxygenation level-dependent (BOLD) signal changes (t-values) were found in the bilateral anterior cingulate cortex (ACC) or (b) posterior cingulate cortex. (TIF) [file pone.0215023.s004.TIF]

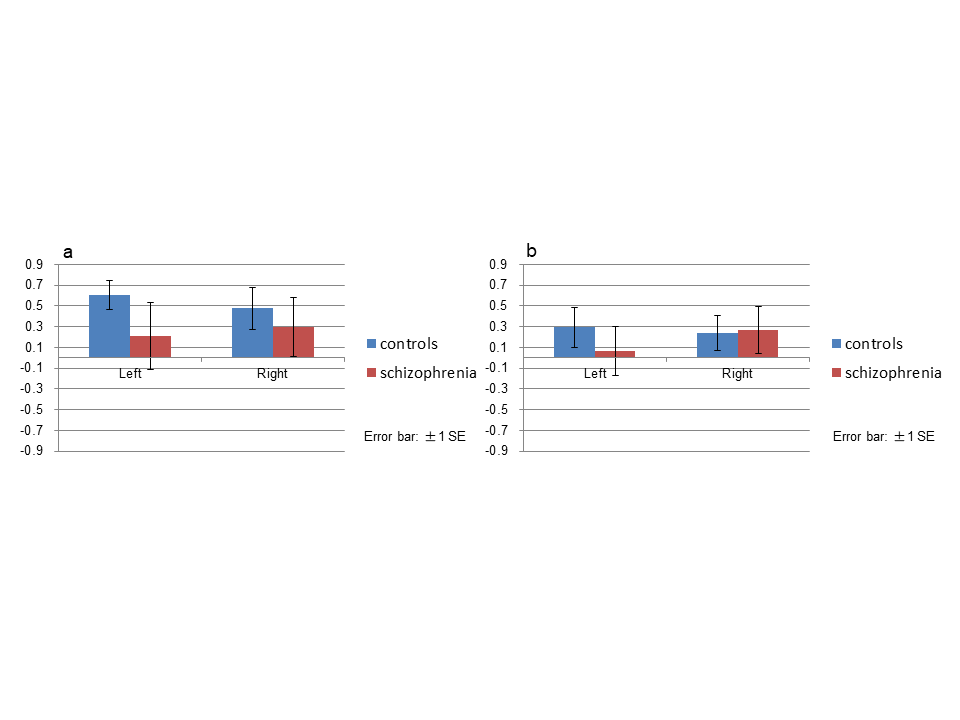

Supplement: S5 Fig — No significant differences were found between patients and controls in the superior temporal gyrus (a) and Heschl’s gyrus (b). Blood oxygenation level-dependent (BOLD) signal changes (t-values) in the left HSC in ROI analysis significantly correlated with MMN amplitudes at the Fz site in patients. (TIF) [file pone.0215023.s005.TIF]

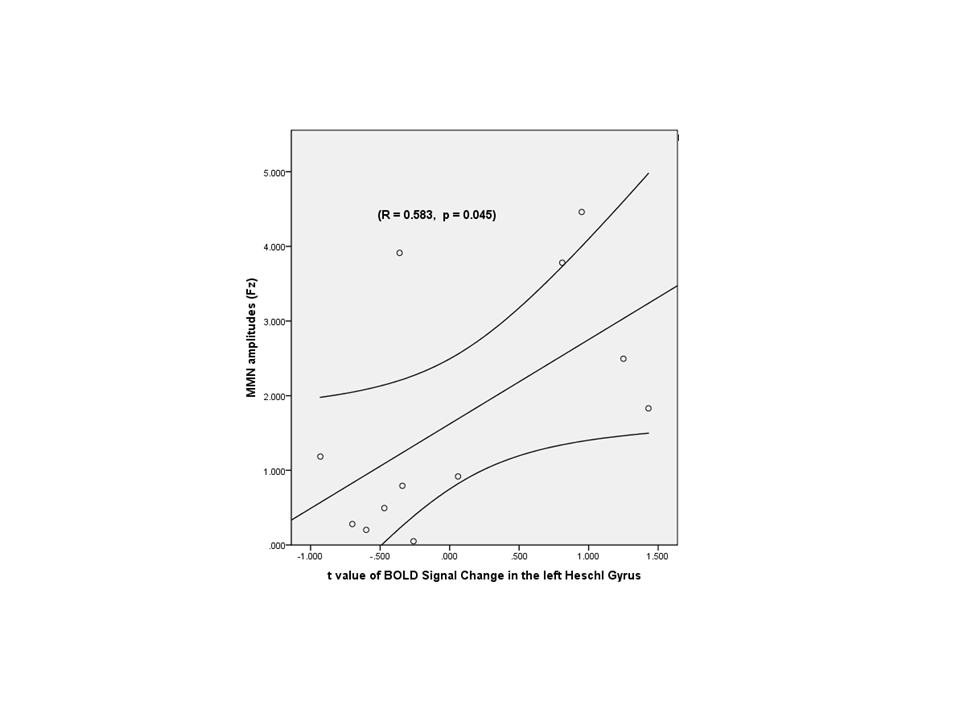

Supplement: S6 Fig — Blood oxygenation level-dependent (BOLD) signal changes (t-values) in the left HSC in ROI analysis significantly correlated with MMN amplitudes at the Fz site in patients. (TIF) [file pone.0215023.s006.TIF]

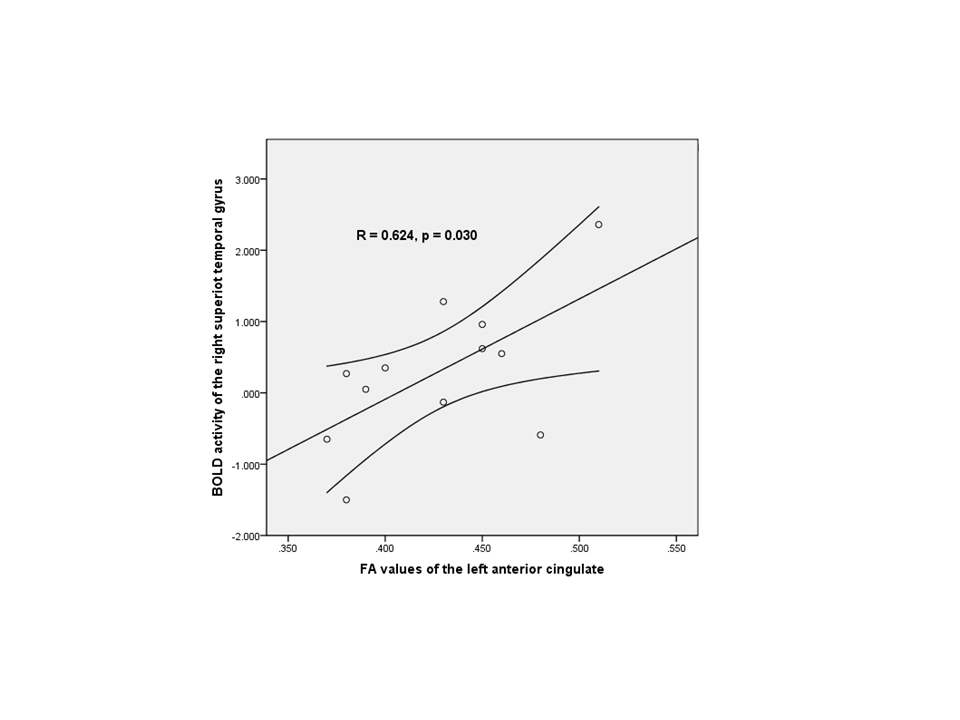

Supplement: S7 Fig — FA values in the left ACC significantly correlated with blood oxygenation level-dependent (BOLD) signal changes in the right STG of patients. (TIF) [file pone.0215023.s007.TIF]

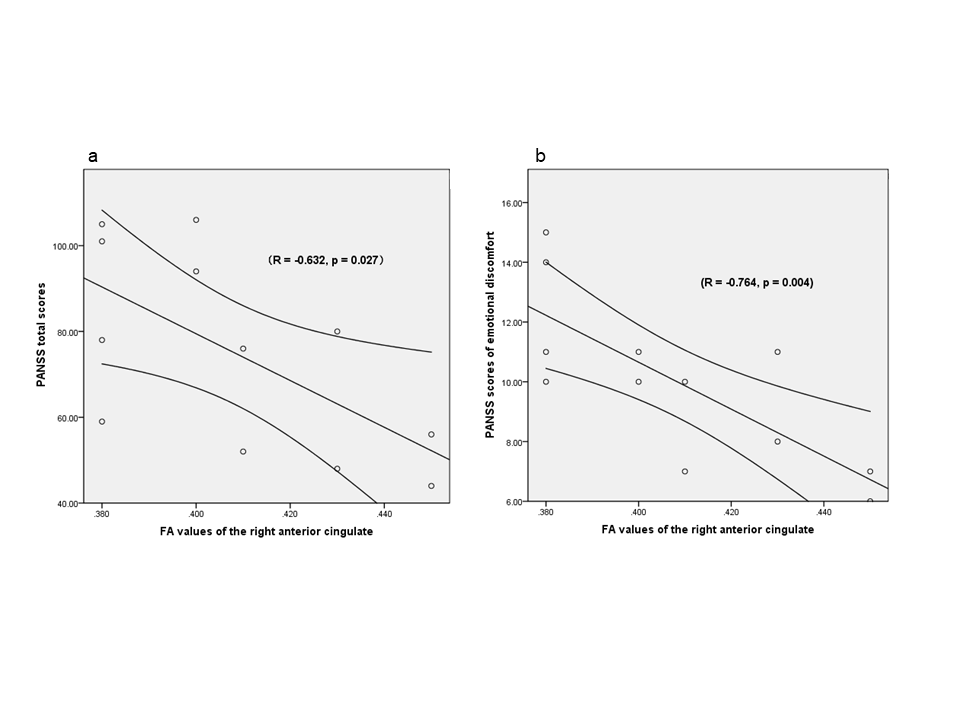

Supplement: S8 Fig — (a) FA values in the right ACC significantly correlated with the Positive and Negative Syndrome Scale (PANSS) total scores and (b) Emotional Discomfort subtotal score as classified by Bell’s classification. (TIF) [file pone.0215023.s008.TIF]
